# Liver cancer initiation requires translational activation by an oncofetal regulon involving LIN28 proteins

**DOI:** 10.1172/JCI165734

**Published:** 2024-06-13

**Authors:** Meng-Hsiung Hsieh, Yonglong Wei, Lin Li, Liem H. Nguyen, Yu-Hsuan Lin, Jung M. Yong, Xuxu Sun, Xun Wang, Xin Luo, Sarah K. Knutson, Christina Bracken, George Q. Daley, John T. Powers, Hao Zhu

**Affiliations:** 1 Children’s Research Institute, Departments of Pediatrics and Internal Medicine, Center for Regenerative Science and Medicine, Simmons Comprehensive Cancer Center, University of Texas Southwestern Medical Center, Dallas, Texas, USA.; 2 Center for Life Sciences, School of Life Sciences, Yunnan University, Kunming, Yunnan, China.; 3 Redona Therapeutics, Watertown, Massachusetts, USA.; 4 Department of Biological Chemistry and Molecular Pharmacology, Harvard Medical School, Boston, Massachusetts, USA.; 5 Department of Pediatrics, Dell Medical School, The University of Texas at Austin, Austin, Texas, USA.

**Keywords:** Hepatology, Liver cancer, Translation

## Abstract

It is unknown which posttranscriptional regulatory mechanisms are required for oncogenic competence. Here, we show that the LIN28 family of RNA-binding proteins (RBPs), which facilitate posttranscriptional RNA metabolism within ribonucleoprotein networks, is essential for the initiation of diverse oncotypes of hepatocellular carcinoma (HCC). In HCC models driven by *NRAS^G12V^/Tp53*, *CTNNB1/YAP/Tp53*, or *AKT/Tp53*, mice without *Lin28a* and *Lin28b* were markedly impaired in cancer initiation. We biochemically defined an oncofetal regulon of 15 factors connected to LIN28 through direct mRNA and protein interactions. Interestingly, all were RBPs and only 1 of 15 was a *Let-7* target. Polysome profiling and reporter assays showed that LIN28B directly increased the translation of 8 of these 15 RBPs. As expected, overexpression of LIN28B and IGFBP1-3 was able to genetically rescue cancer initiation. Using this platform to probe components downstream of LIN28, we found that 8 target RBPs were able to restore *NRAS^G12V^/Tp53* cancer formation in *Lin28a/Lin28b*-deficient mice. Furthermore, these LIN28B targets promote cancer initiation through an increase in protein synthesis. LIN28B, central to an RNP regulon that increases translation of RBPs, is important for tumor initiation in the liver.

## Abstract 2

LIN28 proteins promote tumor initiation in hepatocellular carcinoma through their roles in protein synthesis.

## Introduction

In chronically inflamed tissues subject to field cancerization, tumor initiation occurs repeatedly in different parts of the organ over long time periods. For cancer mutations to drive transformation, cells must be oncogenically competent or able to transform once the requisite mutations are established. We know that not all cells are competent because cancer mutations are often observed in nonmalignant tissues and only some cell types transform when strong cancer mutations are introduced. Oncogenic competence likely requires nonmutational processes that regulate gene expression at epigenetic, transcriptional, and posttranscriptional levels (1–3). Posttranscriptional regulation is known to involve RNA-binding proteins (RBPs), which play diverse and critical roles in RNA editing, localization, stabilization, and translation (4). While these factors are less studied in cancer because they are not as frequently mutated (5), it is clear that dysregulation of RBP expression can promote cancer growth by sustaining cell proliferation, evading apoptosis, avoiding immune surveillance, stimulating angiogenesis, and activating metastasis (6–10). There are many fundamental unanswered questions about RBPs in cancer including the following: how essential are they, and how do they operate on the molecular level?

In this study, we used the LIN28 family of RBPs to explore these questions in animal models. Lin-28 was first identified in *Caenorhabditis* *elegans* as a heterochronic factor that regulates developmental timing (11). In mammals, there are 2 *Lin28* paralogs, *Lin28a* and *Lin28b*, both of which promote stem cell pluripotency, tissue growth, and carcinogenesis (12–21). Both proteins bind and inhibit the biogenesis of the *Let-7* family of tumor-suppressive microRNAs, but through different mechanisms. LIN28B is more concentrated in the nucleus and is thought to block DGCR8-mediated pri- to precursor *Let-7* processing, while LIN28A is localized in the cytoplasm and recruits the 3′ terminal uridylyl transferases (TUTase) named Zcchc11 or Zcchc6 (TUT4 or TUT7) (22–24). LIN28A and TUTases cooperate to uridylate *Let-7* precursors, which targets them for degradation. Both LIN28A and LIN28B are also known to directly interact with thousands of other RNA targets and influence their translation into proteins (23, 25–30). LIN28 proteins, like other RBPs, reside in ribonucleoprotein (RNP) complexes, but it is unclear which individual components of these diverse RNPs are functionally important in cancer.

In general, factors that are required for tumor initiation but not progression are less understood. While factors that regulate progression represent attractive cancer drug targets, improving patient outcomes for cancers that arise from chronic tissue injury would require the suppression of repeated tumor-initiation events. To better understand requirements for RBPs in cancer initiation, we examined the earliest cancer cells. While there is no consensus cell of origin or stem cell for all liver cancer subtypes, a premalignant hepatocellular carcinoma (HCC) progenitor cell was previously shown to be capable of transformation in the setting of diethylnitrosamine (DEN) mutagenesis (31). These progenitors express CD44, aggregate, form suspension colonies in vitro, and can be isolated and transplanted based on these properties. Intriguingly, these progenitors express *Lin28a* and *Lin28b* at high levels. However, the molecular mechanisms dictating the genesis or transformation of these progenitors remain unexplored. We previously found that the deletion of *Lin28a* and *Lin28b* impaired the growth of MYC-induced pediatric hepatoblastomas in vivo, but the absence of *Lin28a/Lin28b* did not completely eliminate their initiation, possibly due to the high levels of *MYC* overexpression in that model (32). It also remains unclear how much LIN28s are required for transformation in the clinically relevant context of chronic liver disease. Furthermore, the identity of the downstream effector mechanisms associated with LIN28 proteins remains unclear. Here, we used versatile mouse models as in vivo platforms for mechanistic dissection of RBPs in cancer initiation. These studies highlight the importance of non–*Let-7*–dependent translational mechanisms downstream of LIN28 and the possibility of using anti-RBP approaches to prevent tumorigenesis.

## Results

### DEN-induced liver cancers are dependent on LIN28s.

**Figure 1:**
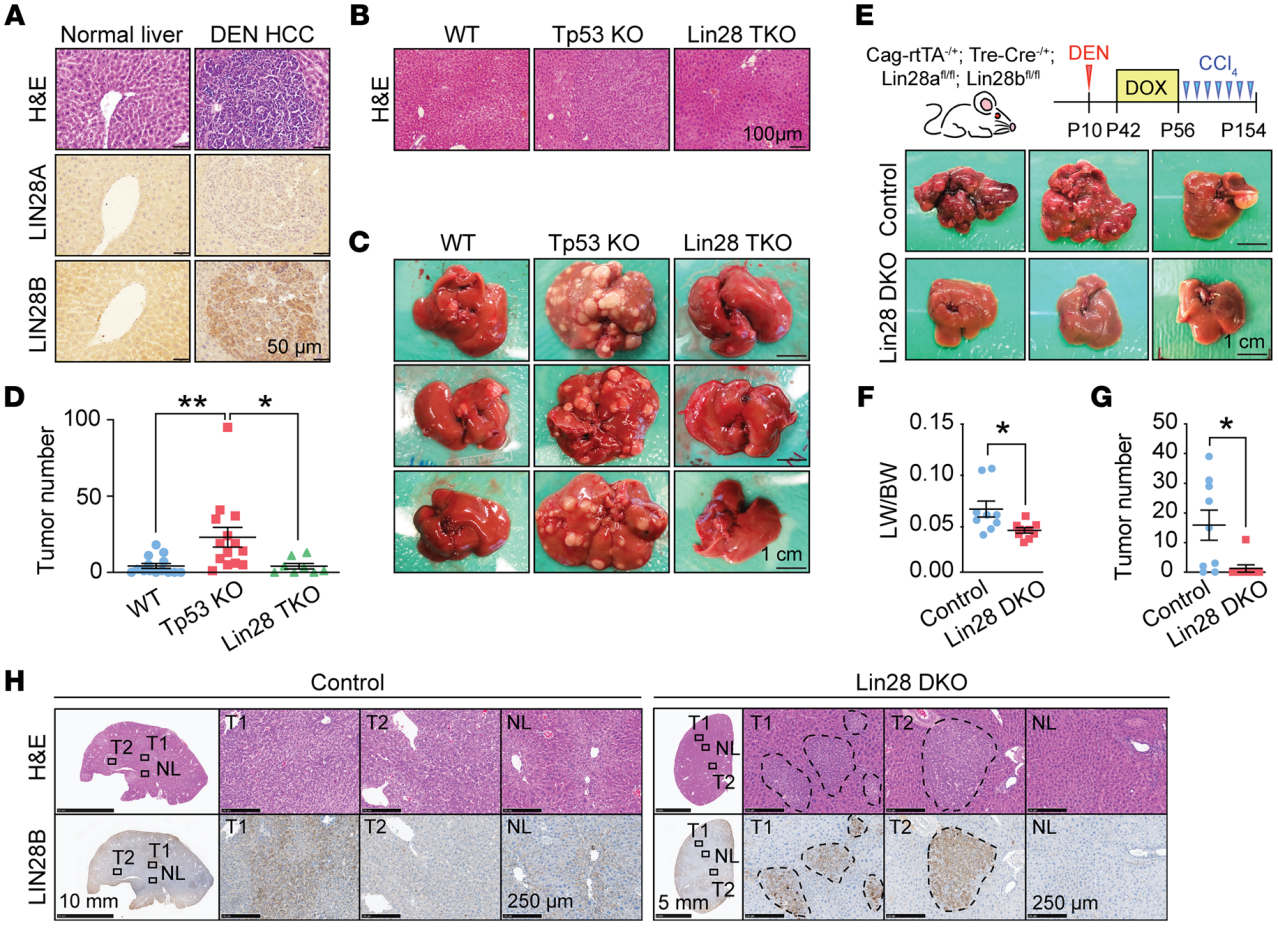
*Lin28*-deficient mice are protected from DEN-driven liver tumorigenesis. (**A**) H&E and IHC of LIN28A and LIN28B of DEN-induced tumor nodules and adjacent tissue from mice treated with DEN for 8 months. Images are representative of 20 individual tumors. Scale bars: 50 μm. (**B**) Histology images show the normal liver architecture of control (*Tp53^fl/fl^*), *Tp53*-KO (*Albumin-Cre; Tp53^fl/fl^*), and *Lin28a/Lin28b/Tp53*-TKO (*Albumin-Cre; Tp53^fl/fl^; Lin28a^fl/fl^; Lin28b^fl/fl^*) mice. Scale bar: 100 μm. (**C**) Representative gross images of WT, *Tp53*-KO, and *Lin28a/Lin28b/Tp53*-TKO livers treated with DEN for 8 months. Scale bar: 1 cm. (**D**) Surface tumor numbers from WT (*n* = 14), *Tp53*-KO (*n* = 14), and *Lin28a/Lin28b/Tp53*-TKO mice (*n* = 8). Each dot represents 1 mouse. (**E**) This schematic shows DEN/CCl_4_ and dox administration. Representative gross images of liver from control (*Cag-rtTA^+/–^; Lin28a^fl/fl^; Lin28b^fl/fl^*; *n* = 9) and *Lin28a/Lin28b*-DKO mice (*Cag-rtTA^+/–^*; *TRE-Cre^+/–^*; *Lin28a^fl/fl^*; *Lin28b^fl/fl^*; *n* = 9) that were subjected to DEN/CCl_4_ administration and dox water. Scale bars: 1 cm. All the images can also be found in Supplemental Figure 1B. (**F** and **G**) Liver-to-body weight ratios (LW/BW) (**F**) and surface tumor numbers (**G**) of control (*n* = 9) and *Lin28a/Lin28b*-DKO (*n* = 9) mice. (**H**) Representative H&E images and LIN28B staining in tumors of control (*n* = 9) and *Lin28a/Lin28b*-DKO mice (*n* = 9). NL, normal liver; T1, tumor number 1; T2, tumor number 2. *P < 0.05; **P < 0.01.

To study tumor initiation in a widely used adult HCC model, we administered DEN to FVB WT mice at 2 weeks of age. At 8 months of age, LIN28A was expressed at low levels, but LIN28B was highly expressed in microscopic hypercellular nodules observed within these livers (Figure 1A). These data suggested that both paralogs could be important for DEN-induced HCC initiation. To determine whether LIN28 proteins are required for tumor initiation, we generated liver-specific *Tp53*-KO mice (*Albumin-Cre*; *Tp53^fl/fl^*) and *Lin28a/Lin28b/Tp53*–triple-KO (TKO) mice (*Albumin-Cre*; *Lin28a^fl/fl^*; *Lin28b^fl/fl^*; *Tp53^fl/fl^*). *Lin28a/Lin28b/Tp53*-TKO mice developed normally, had no obvious whole body or liver-associated phenotypes, and had normal liver histology (Figure 1B). Cohorts of these mice were given DEN at 2 weeks of age and aged for 10 months. Liver-tumor formation was frequent in *Tp53*-KO mice, but was impaired in *Lin28a/Lin28b/Tp53*-TKO mice (Figure 1, C and D); 64% of *Tp53* WT mice, 100% of *Tp53* KO mice, and 62% of *Lin28a/Lin28b/Tp53* TKO mice had gross tumors, and TKO tumors were smaller than tumors from *Tp53*-KO mice.

### DEN mutagen plus CCl_4_ chronic injury–induced HCC also required Lin28s for tumor initiation.

The *Albumin-Cre*–driven model has limitations that prevent a clear understanding of how impactful an anti-LIN28 therapeutic strategy might be in patients with chronic liver damage and HCC. First, it is an embryonic KO and not a model of inducible *Lin28* loss in adults. Second, it is liver specific and does not assess potential extrahepatic toxicities associated with a whole-body *Lin28* loss. Finally, the above DEN-only model did not involve chronic liver injury, which is integral to cirrhosis pathogenesis and the ensuing development of HCC. To address these limitations, we developed an inducible genetic deletion model that would accurately mimic complete LIN28A and LIN28B inhibition in the entire animal. We generated uninducible control (*TRE-Cre*; *Lin28a^fl/fl^*; *Lin28b^fl/fl^*) and inducible whole-body *Lin28a/Lin28b* double-KO (DKO) mice (*CAG-Rtta*; *TRE-Cre*; *Lin28a^fl/fl^*; *Lin28b^fl/fl^*), which allowed us to delete *Lin28a* and *Lin28b* in all cells of the adult mouse upon doxycycline (dox) administration (Figure 1E). At 2 weeks of age (P14), a single dose of DEN was injected to induce liver mutagenesis. At 6 weeks of age, dox was given to all mice for 2 weeks, which caused deletion of *Lin28a/Lin28b* in all cells of experimental mice but had no genetic impact on control mice (Supplemental Figure 1A; supplemental material available online with this article; https://doi.org/10.1172/JCI165734DS1). To introduce chronic injury, we initiated chronic carbon tetrachloride (CCl_4_) twice a week for 12 weeks starting at 10 weeks of age, which caused repeated centrilobular hepatocyte necrosis, accompanied by inflammation and fibrosis.

Almost all gross liver tumors were prevented in the setting of dual *Lin28* deletion (Figure 1, E–G, and Supplemental Figure 1B). Seven of nine control mice developed grossly detectable tumors of the liver surface, while only 1 of 9 *Lin28a/Lin28b*-DKO mice developed tumors (Figure 1G). We asked whether the tumors that arose in the *Lin28a/Lin28b*-DKO background had escaped *Lin28b* deletion, and indeed we detected LIN28B expression in most tumors that formed in the setting of *Lin28a/Lin28b* deletion (Figure 1H). These data indicated that LIN28 expression was critical for cancer development in the DEN alone and in the DEN+CCl_4_ models that integrate mutagenesis, chronic tissue damage, and fibrosis. Next, we asked whether LIN28s might be affecting tumor development through influences on tissue injury or fibrogenesis. In addition to the DEN+CCl_4_ model, we also subjected WT and *Lin28a/Lin28b*-DKO mice to 12 or 20 weeks of long-term CCl_4_ injury without DEN (Supplemental Figure 1C). Even after 20 weeks of CCl_4_, there was no induction of *Lin28a/Lin28b* expression in WT liver tissues (data not shown), and there were no differences in the amount of fibrosis-related gene expression between WT and *Lin28a/Lin28b*-DKO mice (Supplemental Figure 1, C and D). We also examined *Lin28* expression levels in livers from mice fed Western diets for 6 months. While these mice developed fatty liver disease and had increased fibrogenic gene expression (Supplemental Figure 1E), there was no increase in *Lin28a* or *Lin28b* expression (Supplemental Figure 1F). In human livers, *LIN28A* and *LIN28B* overexpression were observed in HCCs, but not in nonmalignant liver tissues from stage 4 fibrosis patients (Supplemental Figure 1, G–J). Collectively, these data suggest that blocking LIN28s impaired cancer formation but did not affect other histologic processes associated with chronic liver disease.

### NRAS^G12V^ activation and Tp53 loss cause a multifocal mixed HCC/cholangiocarcinoma.

It is challenging to dissect the underlying molecular mechanisms associated with mouse models of cancer, in part due to the low-throughput nature of mouse genetics. To dissect LIN28-associated mechanisms in vivo, we needed more rapid models that could also mimic the genetics of the chronic injury models used above. We previously examined the genetic drivers of DEN cancers. Sequencing of 242 of the most commonly mutated genes in human and murine HCCs in 50 individual DEN tumors (33) identified recurrent, mutually exclusive mutations in *Egfr* (Phe254Ile), *Hras* (Gln61Arg), and *Braf* (Val637Glu). These data suggested that DEN tumor transformation depends on at least one of these EGFR-RAS-MAPK–activating mutations. In line with these findings, an increase of p-ERK, a core effector of RAS signaling, was detected in DEN tumors and premalignant lesions (Supplemental Figure 2). To determine whether isolated RAS pathway activation and *Tp53* deletion could recapitulate the DEN-induced HCC models, we tested a model of liver cancer driven by *NRAS^G12V^* activation. We used hydrodynamic transfection (HDT) to introduce a transposable vector containing a constitutively active *NRAS^G12V^* into liver-specific *Tp53*-KO mice. This was sufficient to drive multifocal carcinomas within 2–3 weeks (Supplemental Figure 3A) (34, 35). Individual *NRAS^G12V^*-driven tumors contained both HCC and cholangiocarcinoma (CCA) histologies (Supplemental Figure 3B), a mixed entity that is frequently seen clinically. Regions of CCA stained for CK19 and EPCAM, while regions of HCC expressed high levels of IGF2 and AFP, and both histological types were MYC positive (Supplemental Figure 3, B and C). This *NRAS^G12V^/Tp53* model was advantageous because the driver lesions were defined and because the rate of tumorigenesis was shortened from 8 months to under 1 month.

Within *NRAS^G12V^/Tp53* cancers, we found high LIN28B and modest LIN28A expression in a subset of cancer cells, corroborated by quantitative PCR (qPCR) on bulk tumor samples (Supplemental Figure 3, C and D). We also saw increased expression of IGF2BP1, IGF2BP2, and IGF2BP3, oncofetal proteins known to be regulated by the LIN28/*Let-7* pathway (Supplemental Figure 3, C and D). To determine whether the earliest lesions that ultimately gave rise to these tumors expressed LIN28A or LIN28B, we examined the hypercellular lesions that arose shortly after HDT. These small lesions were detected as early as 12 days after HDT near the pericentral veins or in zone 3 of liver lobules (Supplemental Figure 3E). We hypothesized that these small 5 - to 10-cell diameter lesions could contain the cells of origin for this HCC model. Similar to the DEN-induced HCC progenitors, these lesions expressed IGF2BP3 and were LIN28B but not LIN28A positive (Supplemental Figure 3E), suggesting a greater importance for LIN28B in tumor initiation.

**Figure 2:**
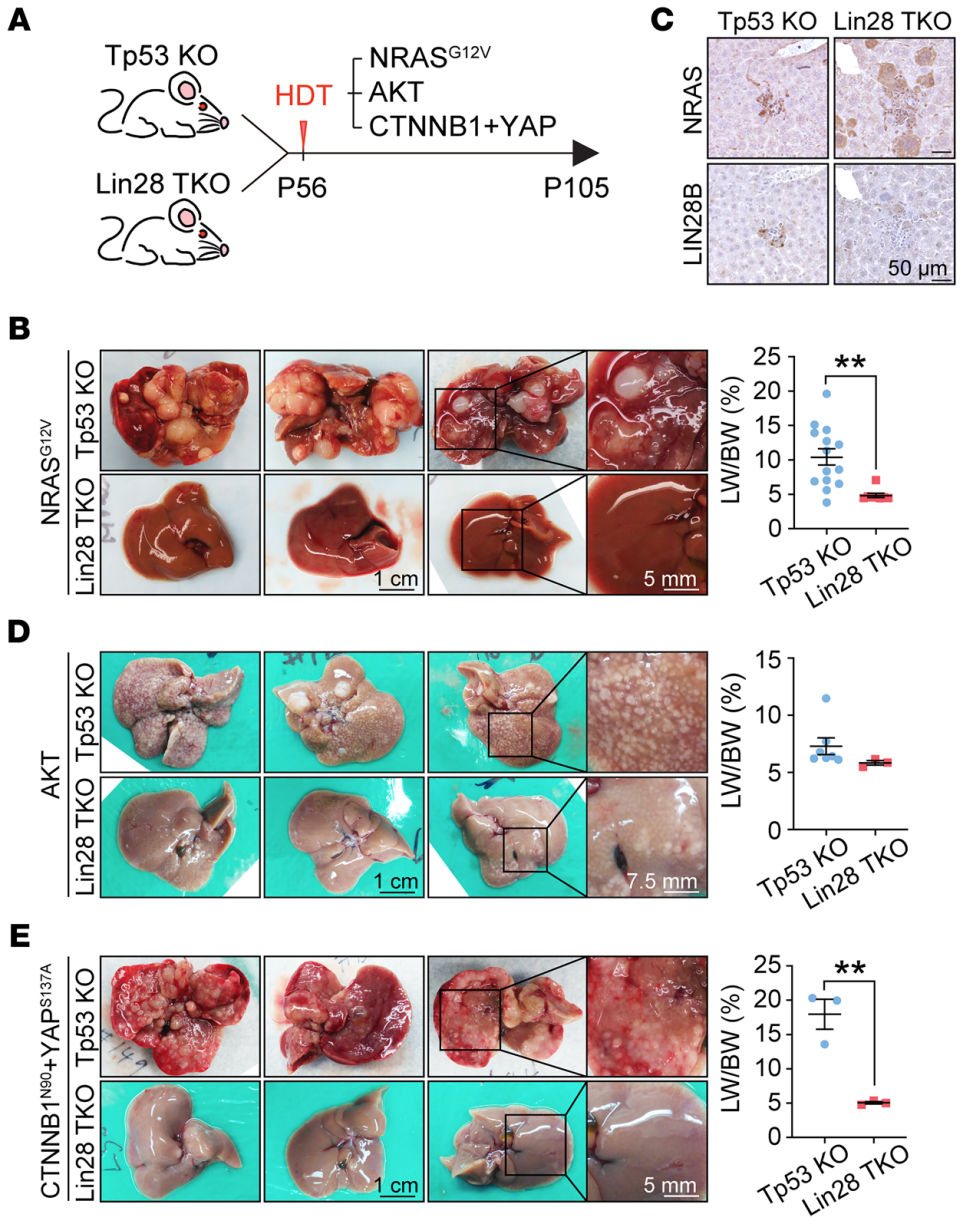
*Lin28a* and *Lin28b* are both required for liver cancer development. (**A**) Schematic for HDT of transposons in *Tp53*-KO and *Lin28a/Lin28b/Tp53*-TKO mice. (**B**) Representative gross images (left) and liver-to-body weight ratios (right) of *Tp53*-KO (*n* = 14) and *Lin28a/Lin28b/Tp53*-TKO (*n* = 8) mice receiving transposons carrying *NRAS^G12V^* for 7 weeks (P105). Scale bars: 1 cm; 5 mm (right panels). (**C**) IHC shows NRAS and LIN28B expression in early lesions of *Tp53*-KO and *Lin28a/Lin28b/Tp53*-TKO mice that had *NRAS^G12V^* injected 2 weeks prior (P70). Scale bars: 50 μm. (**D**) Representative gross images (left) and liver-to-body weight ratios (right) of *Tp53*-KO (*n* = 7) and *Lin28a/Lin28b/Tp53*-TKO (*n* = 3) mice receiving transposons carrying *AKT* for 7 weeks (P105). Scale bars: 1 cm; 7.5 mm (right panels). (**E**) Representative gross images (left) and liver-to-body weight ratios (right) of *Tp53*-KO (*n* = 3) and *Lin28a/Lin28b/Tp53*-TKO (*n* = 3) mice receiving transposons carrying *CTNNB1^N90^* and *YAP^S137A^* for 7 weeks (P105). Scale bars: 1 cm; 5 mm (right panels). **P < 0.01.

To determine whether LIN28 proteins are also required for *NRAS^G12V^/Tp53*-specific liver cancer development, we generated the same *NRAS^G12V^* activation model in the context of liver-specific *Lin28a/Lin28b/Tp53-*TKO mice (Figure 2A). Single *Tp53*-KO mice succumbed to tumorigenesis within 7 weeks, but *Lin28a/Lin28b/Tp53*-TKO mice did not develop any cancers even 3 months after HDT (Figure 2B). This demonstrated an even greater requirement for LIN28 proteins in the *NRAS^G12V^/Tp53* model when compared with the DEN model. To confirm that HDT plasmids were successfully delivered, we showed that NRAS^G12V^ was expressed in the nonmalignant livers of *Lin28a/Lin28b/Tp53*-TKO mice (Figure 2C). As expected, LIN28B expression was observed in *Tp53*-KO but not in *Lin28a/Lin28b/Tp53*-TKO mice (Figure 2C). These results showed that mice without LIN28s are almost completely protected from tumor development in this model, suggesting that LIN28s are required for oncogenic competence and HCC initiation. To determine whether this was an *NRAS^G12V^*-specific effect, we also performed similar experiments with *CTNNB1^N90^*+*YAP^S127A^*+*Tp53*-KO and *AKT*+*Tp53*-KO HDT models (Figure 2, D and E). HCC formation, though not completely blocked, was dramatically reduced in these other models. These observations suggested that LIN28 proteins are critical gatekeepers for transformation driven by multiple oncogenic pathways.

### Using Lin28-deficient mouse models to define bypass and rescue pathways.

**Figure 3:**
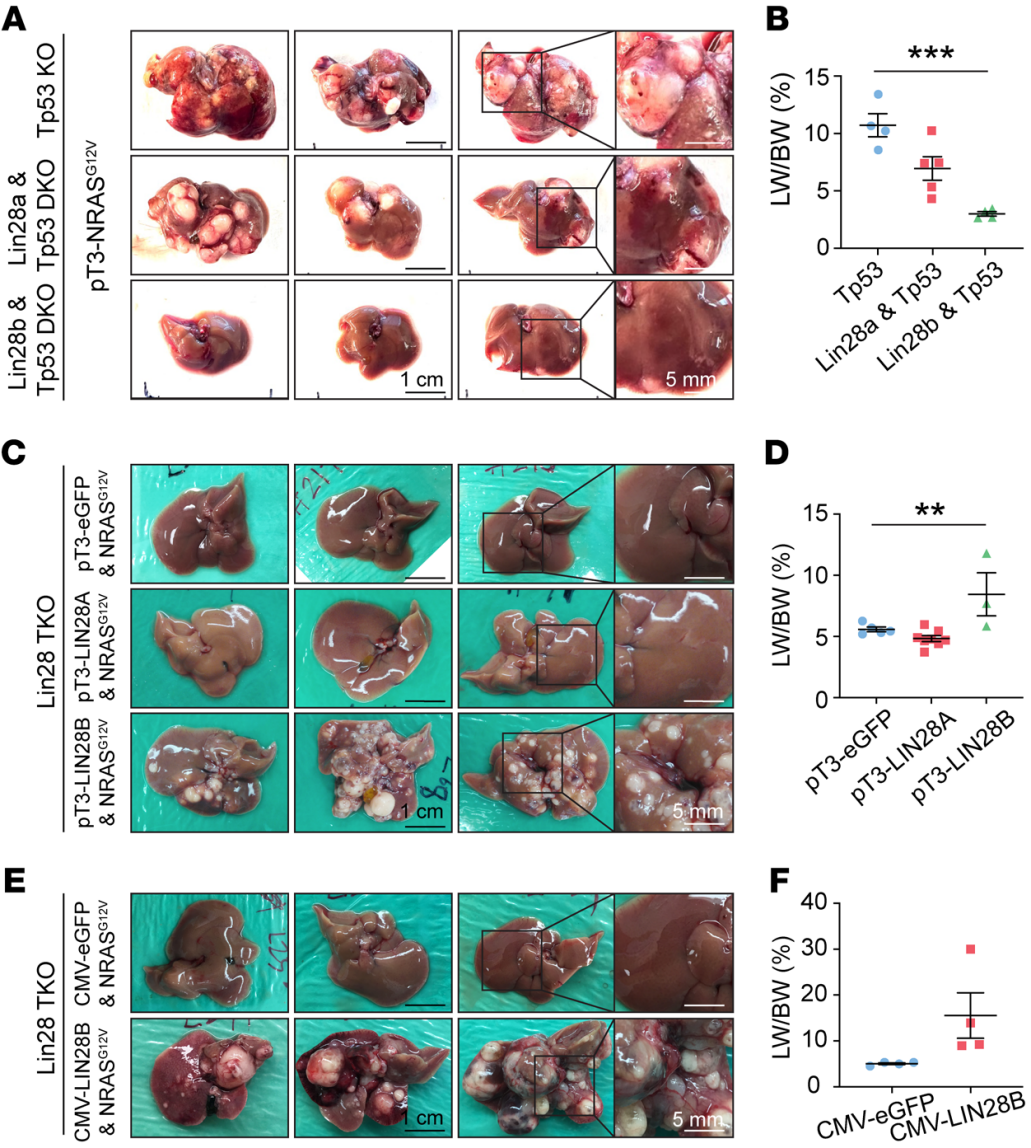
*Lin28b* is indispensable for *NRAS^G12V^/Tp53-*driven liver cancer development. (**A**) Representative gross images of *Tp53-KO* (*Albumin-Cre; Tp53^fl/fl^*; *n* = 4), *Lin28a/Tp53*-DKO (*Albumin-Cre*; *Tp53^fl/fl^*; *Lin28a^fl/fl^*; *n* = 5), and *Lin28b/Tp53*-DKO (*Albumin-Cre*; *p53^fl/fl^*; *Lin28b^fl/fl^*; *n* = 4) mice that received *NRAS^G12V^* by HDT for 7 weeks. Scale bars: 1 cm; 5 mm (right panels). (**B**) Liver-to-body weight ratios for **A**. One-way ANOVA was performed. (**C**) Representative gross images of *Lin28a/Lin28b/Tp53*-TKO mice that received *NRAS^G12V^* in combination with *pT3-eGFP* (*n* = 5), *pT3-LIN28A* (*n* = 8), or *pT3-LIN28B* (*n* = 3) for 7 weeks. Scale bars: 1 cm; 5 mm (right panels). (**D**) Liver-to-body weight ratios for **C**. One-way ANOVA was performed. (**E**) Representative gross images of *Lin28a/Lin28b/Tp53*-TKO mice that received *NRAS^G12V^* combined with *pCMV-eGFP* (*n* = 4) or *pCMV-LIN28B* (*n* = 4) for 7 weeks. Scale bars: 1 cm; 5 mm (right panels). (**F**) Liver-to-body weight ratios for **E**. **P < 0.01; ***P < 0.001.

The rapid carcinogenesis and the stark phenotypic difference between *Tp53*-KO and *Lin28a/Lin28b/Tp53*-TKO mice facilitated mechanistic dissection in vivo. To first determine whether *Lin28a* or *Lin28b* was the more critical component, we genetically segregated mice into ones that had either *Lin28a* or *Lin28b* deletion but not both along with *Tp53* deletion (*Albumin-Cre*; *Lin28a^fl/fl^*; *Tp53^fl/fl^* or *Albumin-Cre*; *Lin28b^fl/fl^*; *Tp53^fl/fl^*). After *NRAS^G12V^* activation in each of these models, we saw reduced tumorigenesis in both *Lin28a/Tp53*- and *Lin28b/Tp53*-DKO models (Figure 3, A and B), but neither model showed the degree of tumor prevention that was seen when both paralogs were deleted. This indicated that each gene was partially responsible for tumor formation, with *Lin28b* having a larger effect. We then asked whether overexpression of a single *Lin28* paralog in tandem with *NRAS^G12V^* activation could rescue the *Lin28a/Lin28b/Tp53*-TKO model. *LIN28B* overexpression was able to rescue HCC (Figure 3, C and D). In contrast, neither *GFP* nor *LIN28A* overexpression was able to rescue HCC. The observation that *LIN28A* did not rescue was consistent with the lack of *LIN28A* expression in early cancer lesions. These results indicated *LIN28A* was required but not sufficient to initiate *NRAS^G12V^/Tp53* liver cancers.

To determine whether *LIN28B* was necessary for cancer maintenance in addition to initiation, we also examined survival in *Lin28a/Lin28b/Tp53*-TKO mice with either transient or continuous overexpression of *LIN28B*. We injected *pT3-NRAS^G12V^* but not *LIN28B* into *Tp53*-KO mice as the control HCC model. This experiment resulted in cancers with ongoing endogenous *Lin28a/Lin28b* expression since the *Tp53*-KO mice are WT for *Lin28a* and *Lin28b*. Transient *Lin28b* overexpression in *Lin28a/Lin28b/Tp53*-TKO mice was achieved with a *LIN28B* plasmid without transposon integration sequences (*pcDNA3.1-CMV-hLIN28B*). Continuous *LIN28B* overexpression in *Lin28a/Lin28b/Tp53*-TKO mice was achieved with a *LIN28B* plasmid with transposon integration sequences (*pT3-LIN28B*). We reasoned that the lack of transposon integration would allow for initiation, but there would be no persistent *LIN28B* to drive long-term tumor growth after initiation. The first observation was that *NRAS^G12V^* plus transient *LIN28B* overexpression in *Lin28a/Lin28b/Tp53*-TKO mice could lead to lethal tumors (Figure 3, E and F) with survival similar to that of the control model (Supplemental Figure 4A), indicating that only a brief burst of *LIN28B* expression was needed to initiate HCC. Two months after HDT, no LIN28B protein expression was seen in the transient rescue group (Supplemental Figure 4B), supporting the observation that LIN28B is required only for initiation. The second major observation is that continuous overexpression of LIN28B did not lead to more aggressive tumor progression compared with the other 2 models, suggesting the lack of a maintenance requirement for LIN28B. We also tested this in vitro by perturbing *LIN28B* expression in Huh7 and SNU308 cells. *LIN28B* knockdown in both Huh7 and HCC53N cells showed no influence on cell proliferation (Supplemental Figure 4, C and D), and overexpression in SNU308 did not cause a marked increase in proliferation (Supplemental Figure 4E). Together, these data indicate that *LIN28B* is not required for tumor maintenance in some HCCs exhibiting LIN28B overexpression.

We then used this in vivo system to define the functional importance of LIN28B’s mRNA targets. By exploiting the difference between the *Tp53*-KO and the *Lin28a/Lin28b/Tp53*-TKO mice, we attempted to identify genetic suppressors of the *Lin28a/Lin28b* deletion phenotype. To determine whether known targets of LIN28A/LIN28B would be able to rescue cancer initiation in *Lin28a/Lin28b*-deficient mice, we tested *Igf2bp1*, *Igf2bp2*, and *Igf2bp3*. Each of these genes is highly expressed in embryos and in human HCCs but not in adult livers (Supplemental Figure 3D and Supplemental Figure 5A) and thus they are considered “oncofetal” RBPs (36, 37). *Igf2bp* mRNAs are known to be stabilized by LIN28 binding and downregulated by *Let-7* (38). We found that the coinjection of either *Igf2bp1*, *Igf2bp2*, or *Igf2bp3* in tandem with *NRAS^G12V^* efficiently rescued cancers in *Lin28a/Lin28b/Tp53*-TKO mice (Supplemental Figure 5B). We reasoned that because these downstream effectors could “suppress” the TKO phenotype, other molecular effectors might also be revealed in a similar way.

### LIN28s bind to RBPs and their mRNAs in an oncofetal regulon.

**Figure 4:**
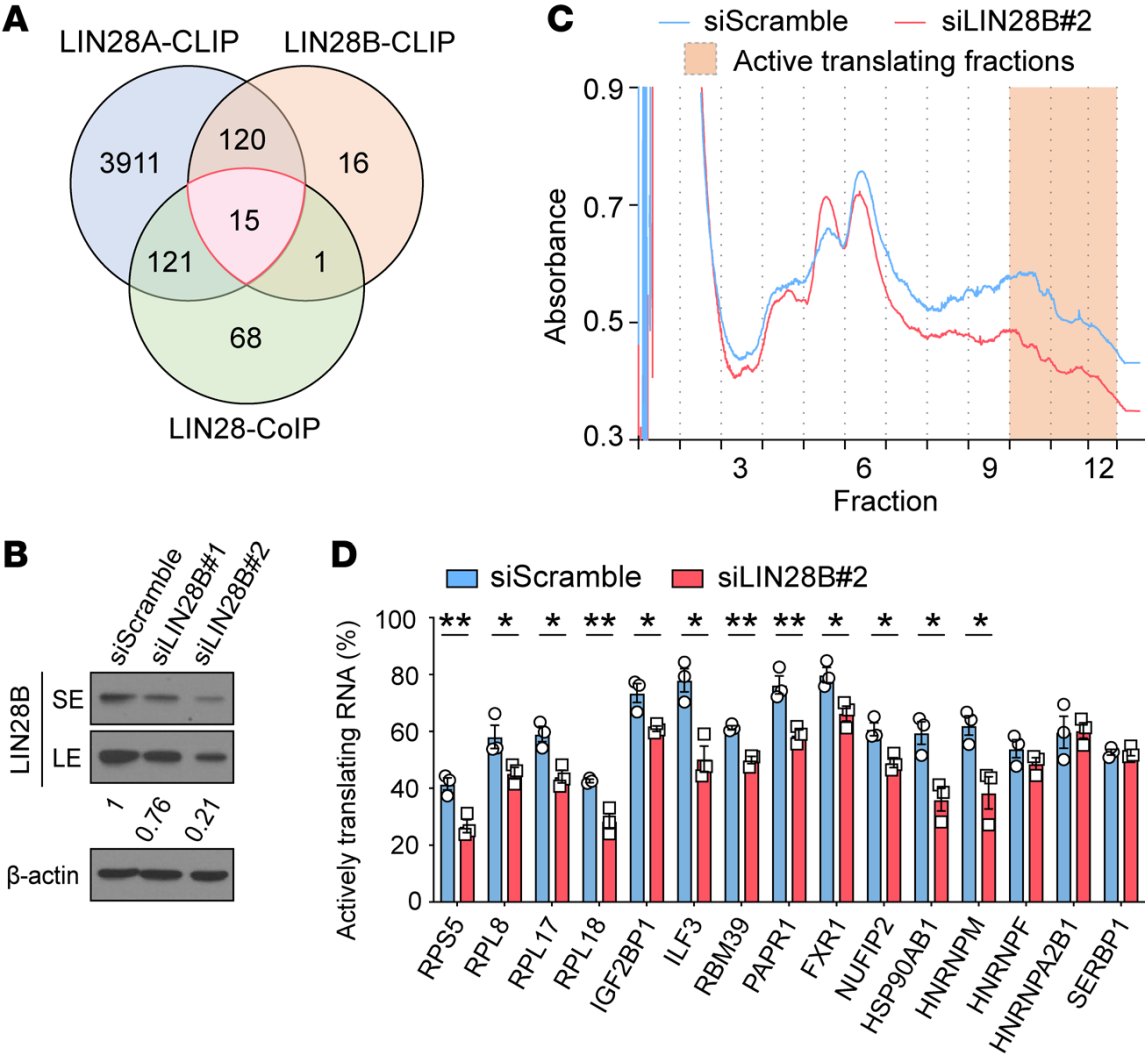
LIN28B promotes the translation of RBP target mRNAs. (**A**) Venn diagram shows 15 factors that bind to LIN28 proteins as both proteins and mRNAs. See gene names in Supplemental Tables 1–4. (**B**) WB analysis shows LIN28B expression 48 hours after siRNA treatment in Huh7 cells. SE, short exposure; LE, long exposure. The numbers below the boxes show relative intensity. (**C**) Polysome profiling shows total translational activity in Huh7 cells with *LIN28B* siRNA knockdown compared with control siRNA–treated cells. The graph shows a representative profile from 3 replicate experiments. (**D**) RT-qPCR analysis of LIN28 targets show the percentage of active translating mRNA fraction in control and *LIN28B* knockdown Huh7 cells. Data include 3 biological replicates. *P < 0.05; **P < 0.01.

To biochemically define mechanisms by which LIN28s promote cancer, we investigated RBP networks involving LIN28 proteins. We hypothesized that there is a LIN28-associated RNA regulon or a group of functionally related mRNAs and proteins whose expression is coordinated through direct interactions. LIN28s interact with thousands of mRNA targets, so we sought to identify the subset of these interactions that might have a larger functional impact. In addition, LIN28 proteins have protein cofactors that likely augment LIN28 functionality. We reasoned that LIN28s could enhance the translation of mRNAs that ultimately become protein cofactors in a feed-forward loop. We sought to identify cofactors that also interact with their own mRNA transcripts by integrating LIN28-mRNA and LIN28-proteomic interaction data sets. We performed LIN28 co-IP and mass spectrometry (MS) on lysates from embryonic stem cells and leukemia cell lines with high levels of LIN28A or LIN28B expression (Supplemental Table 1). We then analyzed published eCLIP data for LIN28A and LIN28B from embryonic stem cells and HEK293T cells (Supplemental Tables 2 and 3) (38–41). This eCLIP data identified mRNAs bound and regulated by LIN28. The intersection of these protein and mRNA interaction data sets revealed 15 factors that bind to LIN28A or LIN28B as both proteins and mRNAs (Figure 4A and Supplemental Table 4). Using Western blotting (WB) and RNA IP qPCR (RIP-qPCR), we confirmed that all 15 targets interacted with LIN28B as mRNAs and proteins (Supplemental Figure 6, A and B). Interestingly, all 15 were RBPs or ribosomal proteins (Supplemental Table 5) and 14 of 15 targets were not likely to be regulated by *Let-7* because they do not have *Let-7*–binding sites in their 3′ UTRs based on TargetScan (https://www.targetscan.org/vert_80/) analysis (only *Igf2bp1* is *Let-7* regulated). According to TCGA transcriptomic data, all 15 were overexpressed in human HCCs as compared with nonmalignant liver tissues (Supplemental Figure 6C), supporting oncogenic functionality for this RNA regulon.

Given the relationship between LIN28s and these RBPs, we hypothesized that LIN28s might directly regulate the stability or expression of their mRNAs. Three possible mechanisms of LIN28 regulation were tested: (a) transcriptional activation, (b) mRNA stabilization, and (c) translational enhancement. To distinguish among these, we performed *LIN28B* loss- and gain-of-function experiments in 2 human liver cancer cell lines that do and do not express *LIN28B* (Huh7 and SNU308, respectively) (Figure 4B and Supplemental Figure 7A). Since LIN28B has been reported to act as a transcription factor and is expressed in the nucleus (42, 43), we asked whether these targets were transcriptionally regulated by LIN28B. Neither *LIN28B* siRNA knockdown nor retroviral overexpression influenced target mRNA levels (Supplemental Figure 7, A and B). Second, we tested the idea that LIN28B could increase target mRNA stability through direct interactions, as suggested previously (38, 44). To examine mRNA stability, we inhibited transcription with actinomycin D after *LIN28B* knockdown or overexpression. These perturbations did not change relative mRNA levels for any target except for *SERBP1* (Supplemental Figure 7, C and D). These data provided little evidence for direct LIN28B effects on mRNA transcription or stabilization.

### LIN28B promoted the translation of target genes through protein-mRNA interactions.

Next, we tested to determine whether LIN28B regulates the translation of some of these targets. Previously, it was shown that LIN28 proteins bring mRNAs to polysomes, resulting in increased translational efficiency (25, 41, 45–47). To more broadly identify mRNA targets whose translation is increased with the presence of LIN28B, we performed polysome analysis of control and *LIN28B* siRNA cell lines. *LIN28B* knockdown in Huh7 resulted in an overall reduction of polysome abundance (Figure 4C). After *LIN28B* knockdown, 12 of the 15 oncofetal regulon mRNAs (*RPL8*, *RPL17*, *RPL18*, *RPS5*, *ILF3*, *IGF2BP1*, *RBM39*, *PARP1*, *HSP90AB1*, *FXR1*, *NUFIP2*, *HNRNPM*) redistributed to less active polysome fractions, indicating reduced association with actively translating ribosomes (Figure 4D). Three targets (*HNRNPF*, *HNRNPA2B1*, *SERBP1*) showed no change in polysome distribution (Figure 4D), indicating that not all of LIN28B’s interacting mRNAs are regulated from a translational standpoint.

**Figure 5:**
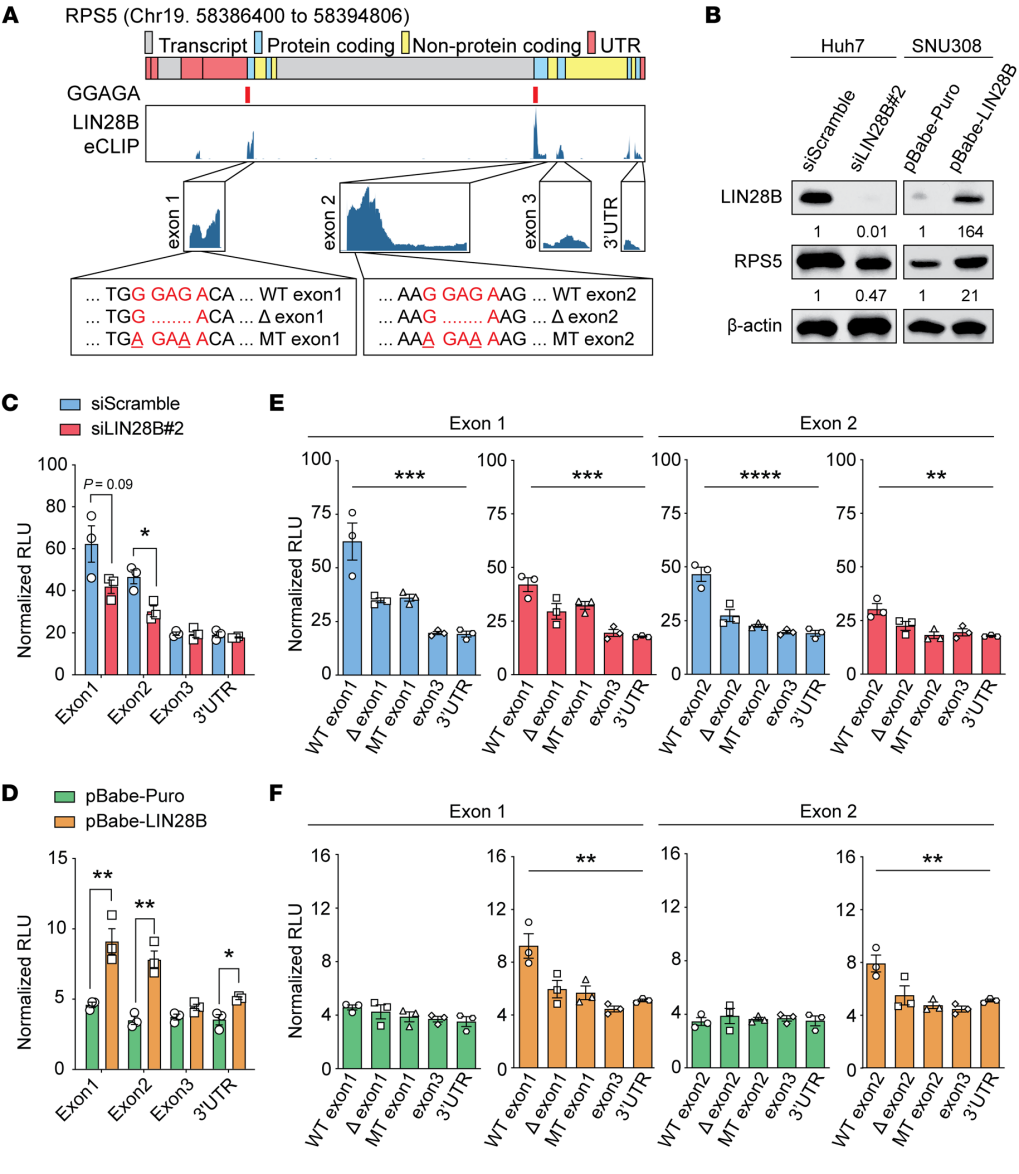
LIN28B regulates *RPS5* translation through direct mRNA binding. (**A**) eCLIP data show LIN28B-binding regions on *RPS5* mRNA. Red marks under the gene indicate the location of consensus LIN28B-binding motifs (GGAGA). Deletion (Δ) and mutation (MT) of consensus motifs were introduced to prevent LIN28B binding. Exon 1, 2, 3, and 3′ UTR sequences were inserted into the Renilla 3′ UTR region. (**B**) WB analysis of RPS5 and LIN28B in Huh7 and SNU308 cell lines. Numbers below the boxes show relative intensities. (**C**) Renilla luciferase activity promoted by *RPS5* exon 1, exon 2, exon 3, and 3′ UTR reporters in *LIN28B* siRNA knockdown (*n* = 3) versus control Huh7 cells (*n* = 3). (**D**) Renilla luciferase activity promoted by RPS5 exon 1, exon 2, exon 3, and 3′ UTR reporters in LIN28B overexpression (*n* = 3) versus control SNU308 cells (*n* = 3). (**E** and **F**) Renilla luciferase activity promoted by WT *RPS5* sequences compared with deletion and mutation containing reporters in control (blue) and *LIN28B* siRNA knockdown (red) Huh7 cells (*n* = 3) (**E**) and in control overexpression (green) and *LIN28B* overexpression (orange) SNU308 cells (*n* = 3) (**F**). One-way ANOVA was performed. **P < 0.01; ***P < 0.001; ****P < 0.0001.

To further determine whether LIN28B directly regulates target mRNAs found in polysomes, we established dual-luciferase reporter assays to probe translational regulation. For ribosomal protein S5 (*RPS5*) mRNA, we identified direct LIN28B-binding regions through examination of eCLIP-Seq data from HepG2, an HCC cell line (48). LIN28B binds to exons 1 and 2 of *RPS5* mRNA (Figure 5A), so these LIN28B-binding regions were cloned into the renilla luciferase reporter 3′ UTR. Because eCLIP data show minimal LIN28B binding to exon 3 or the 3′ UTR of *RPS5*, we used these as negative control sequences. We transfected these reporters into Huh7, an HCC cell line with high *LIN28B* (Figure 5B). *RPS5* exon 1 and exon 2 reporters showed increased luciferase activity when compared with exon 3 or 3′ UTR reporters; *LIN28B* knockdown specifically reduced luciferase only for exon 1 and exon 2 reporters (Figure 5C). We then introduced the same reporters into SNU308, a liver cancer cell line with little *LIN28B* expression. As expected, we did not see a marked difference between exon 1/2 and exon 3/3′ UTR reporters, presumably because LIN28B was not present to affect translation. Consistent with this, exon 1/2 reporter activity in SNU308 increased after *LIN28B* overexpression (Figure 5D). These data indicated that the presence of LIN28B was critical for increasing the translation of LIN28-responsive reporters. Because LIN28B is known to bind a consensus GGAGA motif (41), we identified such motifs in exon 1 and 2 and generated reporters in which these GGAGA motifs were deleted or mutated. The mutated reporters showed decreased luciferase activity compared with WT reporters in Huh7 cells and SNU308 cells overexpressing *LIN28B*, but not in SNU308 without *LIN28B* expression (Figure 5, E and F). We also observed similar findings for interleukin enhancer-binding factor 3 (*ILF3*) reporters (Supplemental Figure 8, A–F). To more directly demonstrate mRNA binding by LIN28B, we overexpressed FLAG-LIN28B and an equal mixture of luciferase constructs containing the WT or 2 mutant motifs. FLAG-LIN28B pulldown led to the enrichment of luciferase mRNA containing the WT binding motif. Both the deletion and mutant binding motifs were not as enriched (Supplemental Figure 8G). Together, these data show that LIN28B binds to specific sequences within target mRNAs, resulting in increased translation.

### LIN28 target genes can rescue tumorigenesis by increasing protein synthesis in Lin28-deficient mice.

**Figure 6:**
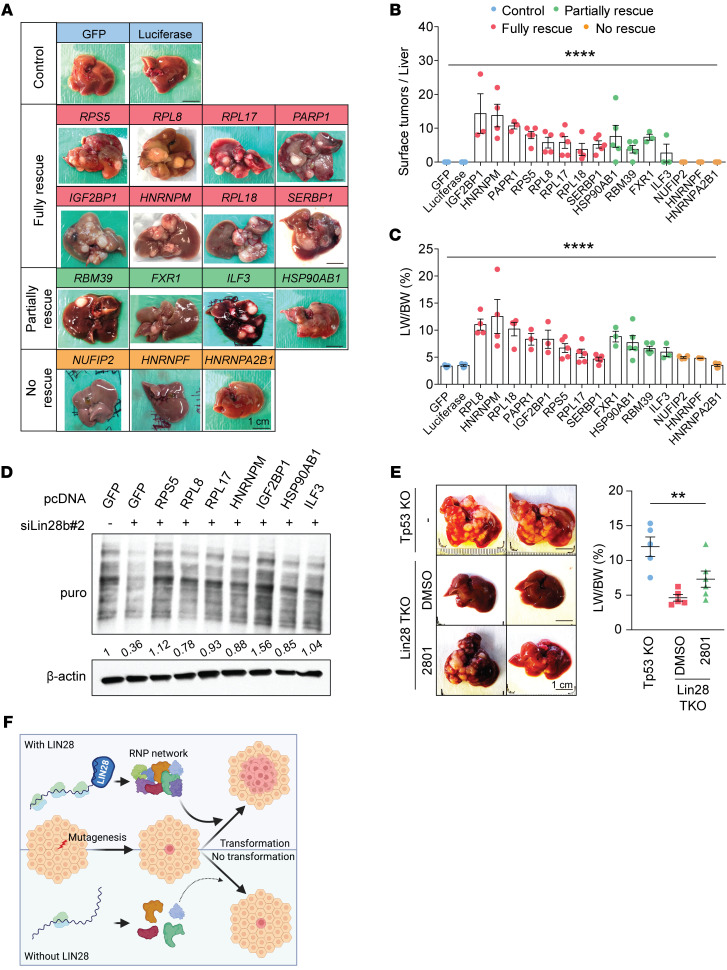
A subset of LIN28’s RBP targets can rescue tumorigenesis in *Lin28a/Lin28b/Tp53*-TKO mice in part through increases in protein synthesis. (**A**) Representative gross images of *Lin28a/Lin28b/Tp53*-TKO mice receiving *NRAS^G12V^* in combination with pT3-RBPs (*n* > 3), pT3-eGFP (*n* = 5), or pT3-Luciferase (*n* = 5) for 7 weeks. Scale bars: 1 cm. All the images can also be found in Supplemental Figures 5B and 9. (**B**) Surface tumor number for **A**. One-way ANOVA was performed. (**C**) Liver-to-body weight ratios for **A**. One-way ANOVA was performed. (**D**) WB analysis quantified OP-puro labeling protein in Huh7 cells with Lin28b knockdown plus target overexpression. Number below the box shows relative intensity. (**E**) Representative gross images of *Tp53*-KO (*Albumin-Cre; Tp53^fl/fl^*; *n* = 5) and *Lin28a/Lin28b/Tp53*-TKO (*Albumin-Cre*; *Tp53^fl/fl^*; *Lin28a^fl/fl^*; *Lin28b^fl/fl^*) mice that received *NRAS^G12V^* by HDT for 7 weeks. TKO mice were subjected to BAZ2A inhibitors (*n* = 6) or DMSO (*n* = 5) once per week starting 3 days after HDT. Scale bars: 1 cm. One-way ANOVA was performed. (**F**) Schematic shows the importance of LIN28 proteins for tumor initiation. The image was designed and drawn using BioRender. **P < 0.01; ****P < 0.0001.

We tested the functional importance of these RNA regulon components by attempting to rescue HCC in the *Lin28a/Lin28b/Tp53*-TKO model. *NRAS^G12V^*, SB100, and GFP injected into TKO mice were not able to initiate any tumorigenesis. To ensure that the lack of tumorigenesis was not due to GFP-related effects, we also showed that a construct containing *Luciferase* did not rescue tumorigenesis (*n* = 3; Figure 6A and Supplemental Figure 9). Next, we asked whether overexpressing LIN28 target genes would be able to rescue HCC in TKO mice. To do this, we individually coinjected 15 transposons containing full-length cDNAs of the RBP targets along with *NRAS^G12V^* and SB100 into TKO mice, then harvested livers 7 weeks after HDT. In contrast with GFP or Luciferase, 8 of 15 LIN28 targets could cause full rescues of HCCs in TKO mice (Figure 6A) and 4 of 15 showed partial rescues (Figure 6, B and C, and Supplemental Figure 9). A full rescue was defined as all mice regaining grossly visible tumors on the liver surface, while a partial rescue was defined as only a subset of mice regaining visible tumors. Interestingly, the genes with the strongest rescue effects were also those that were subject to translational regulation by LIN28B (Figure 4D), while 2 of 3 genes (*HNRNPF* and *HNRNPA2B1*) that did not rescue were not translationally affected by LIN28. This use of transposons containing *GFP* or Luciferase also ensured that the rescue of tumorigenesis was not simply due to the random integration of transposons into cancer-driving loci (Figure 6A and Supplemental Figure 9).

We asked how these targets rescued tumorigenesis in the absence of LIN28 proteins. We observed that LIN28 proteins regulated protein synthesis since the loss of *Lin28a/Lin28b* reduced polysome abundance (Figure 4C). In support of a broad influence on protein synthesis, we performed an additional assay to measure global protein synthesis rates. *O*-propargyl-puromycin (OP-puro) is an analog of puromycin that is incorporated into newly translated proteins. OP-puro is used to measure newly synthesized proteins. Cycloheximide was used as a strong control that almost completely inhibited translation. We observed that *LIN28B* siRNA knockdown decreased OP-puro fluorescence signaling in Huh7 cells (Supplemental Figure 10A). WB analysis also showed a similar finding (Supplemental Figure 10B). Just because LIN28B promoted global protein synthesis rates does not mean that this is the means through which cancer initiation is promoted. We sought to determine whether an increase in protein synthesis is sufficient for cancer initiation. One set of findings that supports a role for protein synthesis is that when we rescue tumorigenesis with target gene overexpression in vivo, the corresponding in vitro overexpression of these targets also showed an increase in global protein synthesis (Figure 6D).

To functionally test whether protein synthesis is sufficient to rescue the effects of *Lin28a/Lin28b* loss, we used an orthogonal method to increase protein synthesis in the absence of LIN28 proteins. We enhanced translation through the inhibition of BAZ2A/B proteins. BAZ2A and BAZ2B are chromatin-remodeling proteins that suppress ribosomal protein and ribosomal DNA transcription. Genetic or pharmacological inhibition of BAZ2A/B proteins leads to increased protein synthesis and liver regeneration (49). A small molecule called GSK2801 promotes increases in protein synthesis through bromodomain inhibition of BAZ2A/B proteins (50). GSK2801 rescued tumor development in *Lin28a/Lin28b/Tp53*-TKO mice. Four out of six mice receiving GSK2801 developed visible tumors on the liver, while 0 of 4 vehicle-treated TKO livers developed tumors (Figure 6E). These data show that increases in translation, in part mediated through LIN28, are important for liver tumor initiation caused by *NRAS^G12V^/Tp53*.

## Discussion

The processes that regulate oncogenic competence and tumor initiation are especially important for cancers associated with chronic inflammatory conditions, such as cirrhosis. While LIN28s are also important for pediatric hepatoblastoma progression (32) and HCC cell line growth in vitro (51), the degree to which they are required for initiation of adult HCC in animal models is not clear. Here, we showed that the lack of LIN28A and LIN28B had no impact on liver development or function, but largely abrogated tumor initiation driven by powerful oncogenes such as *NRAS*, *CTNNB1*, *YAP*, and *AKT*. The phenotypic magnitude of these observations, paired with the fact that LIN28 proteins are dispensable in adults, suggests that they could be attractive targets for liver cancer prevention. It is fortuitous that these particular requirements for oncogenic competence are not apparently essential for normal adult physiology or regeneration.

The stark nature of the *Lin28a/Lin28b* deletion phenotype made it easier to dissect the underlying molecular mechanisms associated with LIN28-driven cancers. LIN28s are well known to inhibit *Let-7* miRNA biogenesis and function. However, the non–*Let-7* target genes that exert important oncogenic effects are comparably less investigated. We hypothesized that LIN28s help to organize a ribonuclear protein regulon involving many other RNAs and RBPs that are required for oncogenic competence. We showed that LIN28s interact with a large number of mRNAs and proteins within cancer cells and most of these interactions did not occur through *Let-7*, given the lack of *Let-7*–binding sites within target mRNAs. Instead, LIN28s directly bind the mRNAs of these targets, bring them to polyribosomes, and increase their protein synthesis rates (Figure 6F).

A key question is how might LIN28 promote translation in general, rather than just the translation of its target genes. Interestingly, many of LIN28’s target proteins are involved in ribosome biogenesis and RNA metabolism, suggesting that the targets of LIN28 might themselves exert broad influences on protein synthesis. For example, RPS5, RPL17, RPL18, and RPL8 are each ribosomal components. Because LIN28 increases the protein production of these ribosomal components, there could be a resulting feed-forward loop that increases global protein synthesis. If LIN28B was only binding nontranslation-related proteins that were not involved in protein synthesis, then one would only see a specific increase in the synthesis of those proteins, but not a global increase in protein synthesis. Indeed, a previous study also showed interactions between LIN28B and general protein synthesis machinery in cancer (29). The interactions between LIN28 and ribosomal components are conserved in different cancer types, including neuroblastoma. In these ways, the reactivation of an oncofetal RBP network involving LIN28 proteins is essential for malignant transformation. A major goal in cancer prevention would be to eliminate or target genes that are required for cancer development, but which have no essential physiological functions. Our study highlights a group of oncofetal genes that might be required during embryogenesis, unnecessary in normal adult tissues, but then required again for cancer initiation.

## Methods

### Sex as a biological variable.

For the DEN-induced HCC model and the CCl_4_-induced chronic injury model, sex was not considered as a biological variable. For HDT-induced HCC models, only males were used due to higher and faster rates of cancer development.

### Mice.

*Albumin-Cre* and *Tp53^fl/fl^* mice were purchased from The Jackson Laboratory and backcrossed more than 15 generations into the FVB/N inbred mouse strain to generate *Tp53*-KO (*Albumin-Cre^+/–^*; *Tp53^fl/fl^*) mice. Conditional *Lin28a*/*Lin28b*-DKO mice (*Cag-rtTA^–/+^*; *TRE-Cre^–/+^*; *Lin28a^fl/fl^*; *Lin28b^fl/fl^*) were reported previously (52, 53). *Lin28a/Tp53*-DKO (*Albumin-Cre*; *Tp53^fl/fl^*; *Lin28a^fl/fl^*), *Lin28b/Tp53*-DKO (*Albumin-Cre*; *Tp53^fl/fl^*; *Lin28b^fl/fl^*), and *Lin28a/Lin28b/Tp53*-TKO (*Albumin-Cre*; *Tp53^fl/fl^*; *Lin28a^fl/fl^*; *Lin28b^fl/fl^*) mice were generated by crossing *Tp53* to *Lin28a* and *Lin28b* double-floxed mice. DEN (MilliporeSigma) was diluted in saline and injected intraperitoneally at age P15 at a dose of 25 μg/g. CCl_4_ (MilliporeSigma) was diluted 1:10 in corn oil (MilliporeSigma) and injected once per week. To induce fatty liver disease, mice were fed with Western diet containing 21.1% fat, 41% sucrose, and 1.25% cholesterol by weight (Envigo, TD.120528) and high sugar water (23.1 g/L D-fructose (Sigma-Aldrich, F0127) and 18.9 g/L D-glucose (Sigma-Aldrich, G8270) as described previously (54).

### Antibodies.

Antibodies used were as follows: NRAS (Abcam, AB77392, IHC), p-ERK (CST, 9101, IHC), LIN28B (Proteintech, 16178-1-AP or CST 5422, IHC and WB), LIN28A (CST, 8641, IHC), c-MYC (MilliporeSigma, M4439, IHC), RPS5 (Abcam, AB210745, WB), IGF2BP1 (Abcam, AB166798, IHC), IGF2BP2 (Abcam, AB124930, IHC), IGF2BP3 (Proteintech, 14642-1-AP, IHC), CK19 (DSHB, TROMA-III, IHC), EpCAM (CST, 14452, IHC), and β-actin (CST 4970, WB).

### Cell culture.

Huh7 and SNU308 cells were provided by Helen Hobbs’s lab at University of Texas Southwestern. The HCC53N cell line was previously generated by our lab from FVB mice with *NRAS^G12V^* and *Tp53* deleted liver cancers. Huh7 cells were cultured in DMEM with 10% FBS and penicillin-streptomycin. SNU308 cells were cultured in DMEM with 10% heat-inactivated FBS and penicillin-streptomycin. All cells were cultured at 37°C in a 5% CO_2_ incubator. For transfections, Huh7 cells (2.5 × 10^5^) were transfected with 25 pmol siRNA (Life Technologies) in 6-well plates by using Lipofectamine RNAiMAX and OptiMEM (Life Technologies), as described in the manufacturer’s instructions. To generate SNU308 cells with *LIN28B* overexpression, cells were transfected in a 10 cm dish using Lipofectamine 3000 and OptiMEM, then selected with puromycin for 1 week to establish stable overexpression clones.

### Protein extraction and WB analysis.

Cells were lysed using RIPA lysis buffer supplemented with Complete Protease Inhibitor (Roche) and subsequent 20% amplitude sonication for 5 seconds. Lysates were cleared by 14,000 *g* centrifugation at 4°C for 15 minutes. Equivalent lysates were separated by SDS-PAGE and electro-transferred onto polyvinylidene difluoride membranes (Fisher Scientific). Following blocking in 5% nonfat dry milk in PBST for 30 minutes, membranes were incubated in primary antibodies overnight. Horseradish-peroxidase–conjugated secondary antibodies diluted 1:5,000 in 5% nonfat milk were used. Bands were visualized with SuperSignal West Pico or Femto substrate kits (Thermo Fisher). The following commercial primary antibodies supplemented with 0.02% sodium azide were used for immunoblot analysis: NRAS, LIN28B, RPS5, RPL8 (Abcam, AB155136), RPL17 (Abcam, AB155781), RPL18 (Abcam, AB241988), IGF2BP1, ILF3 (Abcam, AB92355), RBM39 (Sigma-Aldrich, HPA001591), PARP1 (Abcam, AB191217), FXR1 (Abcam, AB155124), SERBP1 (Thermo Fisher Scientific, TA800699), HNRNPF (Invitrogen, PA5-79382), HNRNPM (Invitrogen, MA1-34981), HNRNPA2B1 (Invitrogen, PA5-34939), HSP90AB1 (Invitrogen, MA1-10372), puromycin (Thermo Fisher Scientific, MABE343MI), and β-actin.

### Molecular biology and cloning.

The transposon plasmids used for in vivo tumorigenesis rescue studies were modified from pT3-Ef1ɑ-GW, which was provided by Xin Chen at UCSF (San Francisco, California, USA). Full-length cDNAs including *GFP*, Luciferase, *RPS5*, *RPL8*, *RPL17*, *RPL18*, *SERBP1*, *PARP1*, *IGF2BP1*, *RBM38*, *FXR1*, *ILF3*, *HSP90AB1*, *NUFIP2*, *HNRNPM*, *HNRNPF*, and *HNRNPA2B1* were purchased from Horizon Discovery and used to replace the CcdB gene using the gateway LR recombination reaction (Invitrogen, 11791100). Sequences were then confirmed by Sanger sequencing.

### HDT.

SB100, pT3-*MYC*, and pT3-*CTNNB1* transposon plasmids were obtained from Xin Chen at UCSF. All mice were injected at approximately 6 weeks of age, when their body weights were at least 20 g. HDT plasmids were suspended in 2 mL of saline and administered via tail-vein injection over 7 seconds. A 10:1 mass ratio of combined HDT plasmids to SB100 transposase plasmid was used.

### Histology and IHC.

Tissues were fixed in 4% paraformaldehyde for 16 to 24 hours and embedded in paraffin. Tissue sections were deparaffinized with xylene and rehydrated with a graded series of ethanol (100%, 95%, 80%, and 50% ethanol and distilled water), followed by 2 washes of 5 minutes each in PBS with 0.05% Tween-20 (PBST). Antigen retrieval was performed for 20 minutes in sodium citrate buffer (10 mM at pH 6) at 90–100°C, followed by a wash with PBST for 5 minutes. Tissue sections were then incubated for 10 minutes in 3% (vol/vol) hydrogen peroxide in methanol to block endogenous peroxidase activity. Sections were then washed for 5 minutes in PBST and blocked at 25°C for 1 hour using 2% normal goat serum, 2% BSA, and 0.1% Triton X-100 in PBS. Tissue sections were then incubated in a humidified chamber overnight at 4°C with primary antibody (1/200 in TBST). Sections were subsequently washed with PBST (3× for 5 minutes each) and incubated at 25°C for 1 hour with a secondary antibody. After washing with PBST (3× for 5 minutes each), sections were incubated with ready-to-use streptavidin peroxidase (Lab Vision) for 10 minutes at 25°C and then color was developed using the DAB Kit (Vector Laboratories). Sections were counterstained with hematoxylin. The following primary antibodies were used: NRAS, p-ERK, LIN28B, LIN28A, c-MYC, IGF2BP1, IGF2BP2, IGF2BP3, CK19, and EpCAM.

### RNA extraction and RT-qPCR.

Liver total RNA was isolated using TRIzol reagent (Invitrogen, 15596018) followed by purification using the RNeasy Mini Kit (QIAGEN). For reverse-transcription qPCR (RT-qPCR), cDNA synthesis was performed with 1 μg of total RNA using iScript RT Supermix (Bio-Rad, 1708840) in a total of 20 μl volume per reaction. To measure mRNA expression, each cDNA sample (20 μl) was diluted to 200 μl, and 5 μl was combined with primers and iTaq Universal SYBR Green Supermix (Bio-Rad, 172-5121) in a total of 12 μl volume of reaction. The mRNA levels were normalized to β-actin expression. The following RT-qPCR primers were used: *hLIN28A* (forward: 5′-GAGCATGCAGAAGCGCAGATCAAA; reverse: 5′-TATGGCTGATGCTCTGGCAGAAGT)*, hLIN28B* (forward: 5′-GCCCCTTGGATATTCCAGTC; reverse: 5′-TGACTCAAGGCCTTTGGAAG)*, hGAPDH* (forward: 5′-ATGGCCTTCCGTGTTCCT; reverse: 5′-CAGGCGGCACGTCAGAT), *hVIM* (forward: 5′-TGTCCAAATCGATGTGGATGTTTC; reverse: 5′-TTGTACCATTCTTCTGCCTCCTG), *hCOL1A1* (forward: 5′-GATTCCCTGGACCTAAAGGTGC; reverse: 5′-AGCCTCTCCATCTTTGCCAGCA), *hACTA2* (forward: 5′-AAAAGACAGCTACGTGGGTGA; reverse: 5′-GCCATGTTCTATCGGGTACTTC), *hRPS5* (forward: 5′-ATGACCGAGTGGGAGACAG; reverse: 5′-GCTTTGCGGAAGCGTTTGG), *hRPL8* (forward: 5′-AAGGGCATCGTCAAGGACATC; reverse: 5′-CAGCTCCGTCCGCTTCTTAAA), *hRPL17* (forward: 5′-GAACCCCACGAAATCATGCAA; reverse: 5′-TGAACACGAAGATTGGAACCTC), *hRPL18* (forward: 5′-ATGTGCGGGTTCAGGAGGTA; reverse: 5′-CTGGTCGAAAGTGAGGATCTTG), *hIGF2BP1* (forward: 5′-GCGGCCAGTTCTTGGTCAA; reverse: 5′-TTGGGCACCGAATGTTCAATC), *hILF3* (forward: 5′-AGCATTCTTCCGTTTATCCAACA; reverse: 5′-GCTCGTCTATCCAGTCGGAC), *hRBM39* (forward: 5′-CAATGCTTGAGGCTCCTTACA; reverse: 5′-TCCGTTCCTTACTTTTGCTTCTC), *hPARP1* (forward: 5′-CGGAGTCTTCGGATAAGCTCT; reverse: 5′-TTTCCATCAAACATGGGCGAC), *hFXR1* (forward: 5′-GAGAAGACGGTATGGTTCCATTT; reverse: 5′-AGGCGTTCCATTCTTAGCTGT), *hNUFIP2* (forward: 5′-GGTGAACTAAACGGTAATGCTGG; reverse: 5′-GCTAGTGTCTACAACTTGCTGG), *hSERBP1* (forward: 5′-ATTTGACGACGAATCGGACCC; reverse: 5′-GTTCTTGCGGTCTTTCTGGGA), *hHNRNPF* (forward: 5′-CTGCTCTGTTGAGGACGTG; reverse: 5′-CCTGCCCTCTCTAGTGTAGATG), *hHNRNPM* (forward: 5′-GCGGCGACGGAGATCAAAA; reverse: 5′-CTCATTCTGAGCAGGTCGTTC), *hHNRNPA2B1* (forward: 5′-ATTGATGGGAGAGTAGTTGAGCC; reverse: 5′-AATTCCGCCAACAAACAGCTT), *hACTB* (forward: 5′-AGAAGGATTCCTATGTGGGCG; reverse: 5′-CATGTCGTCCCAGTTGGTGAC), *mAfp* (forward: 5′-CTGGCGATGGGTGTTTAGAA: reverse: 5′-GCCTGAGAGTCCATACTTGTTAG), *mIgf2* (forward: 5′-TACCTCTCAGGCCGTACTT; reverse: 5′-ACTGTCTCCAGGTGTCATATTG), *mLin28a* (forward: 5′-AGGCGGTGGAGTTCACCTTTAAGA; reverse: 5′-AGCTTGCATTCCTTGGCATGATGG), *mLin28b* (forward: 5′-TTTGGCTGAGGAGGTAGACTGCAT; reverse: 5′-ATGGATCAGATGTGGACTGTGCGA), *mIgfbp1* (forward: 5′-ATCAGCCCATCCTGTGGAAC; reverse: 5′-TGCAGCTAATCTCTCTAGCACTT), *mIgfbp2* (forward: 5′-CAGACGCTACGCTGCTATCC; reverse: 5′-CCCTCAGAGTGGTCGTCATCA), *mIgfbp3* (forward: 5′-CGCCCCACTTACAATGGGAG; reverse: 5′-CTGCCGTTTCCGAATCCGT), *mVim* (forward: 5′-ACCGCTTTGCCAACTACAT: reverse: 5′-TTGTCCCGCTCCACCTC), *mActa2* (forward: 5′-GAGAAGCCCAGCCAGTCG; reverse: 5′-ATCTTTTCCATGTCGTCCCAGTTG), *mCol1a1* (forward: 5′-TTCTCCTGGCAAAGACGGACTCAA; reverse: 5′-AGGAAGCTGAAGTCATAACCGCCA), and *mGapdh* (forward: 5′-ACCACAGTCCATGCCATCAC; reverse: 5′-TCCACCACCCTGTTGCTGTA).

### Luciferase assays.

LIN28B-binding sites in the human *RPS5, ILF3, IGF2BP1*, *HSP90AB1*, and *HNRNPM* genes plus approximately 250 bp of flanking sequence were amplified and ligated into the XbaI site of pGL3-control (Promega). Mutagenesis was performed by reamplifying each fragment with primers containing the correct mutations. Mutated fragments were then reintroduced into the pGL3 3′ UTR reporter plasmid using the In-Fusion HD Cloning Kit (Clontech). Twenty-four hours after transfection, cells were lysed and assayed for firefly and renilla luciferase activity using the Dual-Luciferase Reporter Assay System (Promega). Firefly luciferase activity was normalized to renilla luciferase activity to obtain relative luciferase activity. All transfections were performed in triplicate, and 3 experimental trials were performed.

### Polysome profiling.

Sucrose gradients were prepared right before use in Beckman ultracentrifuge tubes as described previously (55). One day prior to the experiment, gradients were allowed to diffuse for 16 hours at 4°C. The next day, 20–40 × 10^6^ Huh7 cells were trypsinized and washed 2× with ice-cold PBS containing 100 μg/mL cycloheximide. After the second wash, PBS was discarded and cell pellets resuspended in 750 μL of Polysome Extraction Buffer (20 mM Tris-HCl [pH 7.5], 100 mM NaCl, 5 mM MgCl_2_, 0.1% NP-40 in distilled water) containing cycloheximide, protease inhibitor cocktail, and RNAse inhibitors. Cells were lysed for 10 minutes on ice and sheared through a 27.5-gauge needle 3–4 times. The lysates were centrifuged at 15,000 *g* for 5 minutes at 4°C and the supernatant lysate RNA concentration was quantified by NanoDrop (Thermo Fisher Scientific). Equal amounts of lysate (500–600 μg RNA) were loaded across all gradients. The gradients were centrifuged at 35,000 *g* for 2 hours at 4°C and run on a fractionator machine (Bio-Rad) to visualize and collect polysome fractions. Each collected fraction was mixed with 3× volume of 100% ethanol and 20 μg glycogen carrier and incubated overnight at −20°C. The next day, fractions were centrifuged at 20,000 *g* for 30 minutes at 4°C to precipitate RNA pellets. Pellets were dried for 20 minutes at room temperature, resuspended in 100 μL Nanopure water (Thermo Fisher Scientific) and 350 μL RNeasy RLT lysis buffer, and loaded onto RNeasy columns (QIAGEN). The RNeasy Kit was used to isolate RNA; then cDNA synthesis and RT-qPCR were performed, and 20 ng of Luciferase mRNA control (Promega, L4561) was added to each fraction prior to RNA extraction to control for variability in total RNA in fractions during RNA isolation and reverse transcription. Fractions associated with fewer than 3 ribosomes were grouped together (poorly translated mRNAs), and fractions with more than 3 ribosomes were grouped together (efficiently translated mRNAs). RT-qPCR was used to quantify mRNA levels in each group. Experiments on all groups were performed in triplicate, and multiple experimental trials were performed.

### MS.

FLAG-LIN28B and associated proteins were immunoprecipitated with ANTI-FLAG M2 affinity agarose beads according to the manufacturer’s protocol (MilliporeSigma, catalog A2220). Coprecipitated proteins were separated on a 4%–20% polyacrylamide gel (Bio-Rad) and visualized using the Bio-Safe Coomassie Stain (Bio-Rad). Multiple bands covering most protein sizes were excised and treated with dithiothreitol and iodoacetamide. Proteins were digested in gel. Resulting peptides were gel extracted and analyzed by liquid chromatography–MS (LC-MS) as described previously (56). Peptide matches were filtered by mass accuracy, tryptic state, XCorr, and confirmed by manual inspection.

### OP-puro labeling.

For puromycin labeling of newly synthesized protein in cells, after 48 hours of siRNA transfection, 1 μM OP-puro was added to cells for 1 hour. For Western quantification, cells were then washed with cold PBS and lysed with RIPA. For immunocytochemistry analysis, cells were fixed in 4% formaldehyde for 10 minutes, then permeabilized with 0.05% Triton X-100.

### Immunofluorescence.

Cells were fixed in 4% PFA for 10 minutes, then permeabilized with 0.5% Triton X-100 for 10 minutes. Cells were then blocked with 5% BSA for 1 hour. After blocking, cells were incubated with primary antibodies overnight. Then cells were washed 3× with PBS, followed by secondary antibody incubation for 1 hour. Nuclei were stained with Hoechst before mounting to a microscope slide. Images were captured using confocal microscopy (Zeiss) and assembled using Fiji ImageJ software (version 2.0.0-rc-43/1.51q). The following antibodies were used: LIN28B, puromycin, anti-mouse Alexa Fluor 488 (Life Technologies, A21131), and anti-rabbit Alexa Fluor 594 (Life Technologies, A21207).

### IP.

To examine the interactions between LIN28B protein and target proteins, FLAG-LIN28B constructs were transfected into HEK-293T cells grown in 6-well plates at 80% confluence. One day after transfection, cells were trypsinized and seeded in 10 cm dishes. Cells were harvested when they reached 80% confluence using Pierce RIPA buffer. Anti-FLAG M2 agarose beads (Sigma-Aldrich, A2220, 25 μl of the slurry beads per sample) were washed with Wash Buffer (Thermo Fisher Scientific, 88828, buffer A supplemented with 200 mM NaCl) added to the extracts and rotated overnight at 4°C. After incubation, beads were washed with Wash Buffer 3× and directly boiled with 1× protein loading buffer for 10 minutes at 95°C. The supernatants of the boiled samples were resolved by SDS-PAGE and analyzed by WB. The following antibodies were used: FLAG (CST, 14793S) and rabbit-IgG (CST, 2729).

### Statistics.

The data in most panels reflect multiple experiments performed on different days using mice derived from different litters. In all experiments, mice were not excluded from analysis after the experiment was initiated unless the mice died. Data and error bars indicate mean and SEM. Statistical tests used are noted in the figure legends. Unless otherwise stated in the methods or figure legends, 2-tailed Student’s *t* tests (2-sample equal variance) were used to test the significance of differences between 2 groups. For time-course–expression experiments, 1-way ANOVA was performed and the significance shown was compared with the initial time point. For experiments involving 2 groups and different time points, 2-way ANOVAs were used, and the significance was compared with the means of each group at different time points. Variation is indicated using SEM and presented as mean ± SEM. Statistical significance was defined as *P* < 0.05.

### Study approval.

All mice were handled in accordance with the guidelines of the Institutional Animal Care and Use Committee at University of Texas Southwestern under protocol APN 2015-101118. For human tissue samples, all patients gave consent under Institutional Review Board STU062013-063 or STU092013-010.

### Data availability.

All TCGA data used in this study were obtained through the NIH National Cancer Institute (NCI) (https://portal.gdc.cancer.gov/). All data supporting the findings are available within the article, supplemental materials, or Supporting Data Values files. Other data can be obtained upon request.

## Author contributions

MHH, YW, and HZ conceived the project, performed the experiments, and wrote the manuscript. JMY, LL, XS, LHN, XW, and YHL assisted with experiments and mouse husbandry. XL performed bioinformatic analyses. SKK and CB contributed to conceptualization of the project. JTP and GQD performed LIN28B co-IP experiments and MS.

## Supplementary Material

Supplemental data

Unedited blot and gel images

Supplemental tables 1-5

Supporting data values

## Version 1 06/14/2024

In-Press Preview

## Version 2 08/01/2024

Electronic publication
