# Supplementary material for: Liver cancer initiation requires translational activation by an oncofetal regulon involving LIN28 proteins
Source: J Clin Invest. 2024 Jun 13;134(15):e165734. doi: 10.1172/JCI165734 (PMC11290964; doi:10.1172/JCI165734)
Supplement: Supplemental data [file jci-134-165734-s146.pdf]

SuppFigure 1.

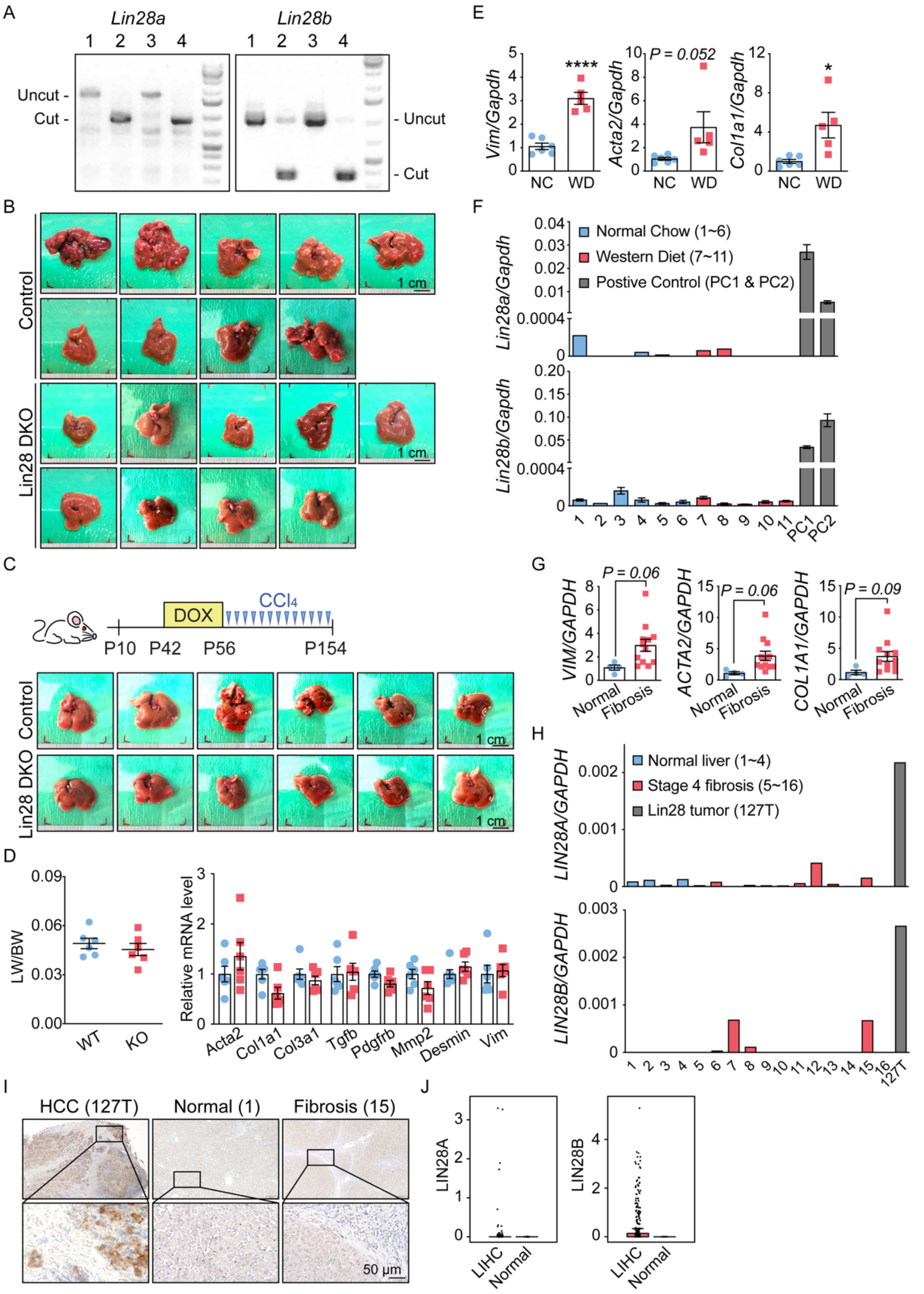

Supplemental Figure 1. LIN28s have no little impact liver regeneration and fibrosis.

(**A**) Genotyping results showing *Lin28* gene deletion of the dox-inducible *Lin28a/Lin28b* DKO system after dox treatment. Column 1: *Tp53* KO mice (*Albumin-Cre; Tp53$^{fl/fl}$*); 2: *Lin28a/Lin28b/Tp53* TKO mice (*Albumin-Cre; Lin28a$^{fl/fl}$; Lin28bb$^{fl/fl}$; Tp53$^{fl/fl}$*); 3: inducible *Lin28a/Lin28b* control mouse (*TRE-Cre; Lin28a$^{fl/fl}$; Lin28bb$^{fl/fl}$*); 4: inducible *Lin28a/Lin28b* DKO mouse (*CAG-Rtta; TRE-Cre; Lin28a$^{fl/fl}$; Lin28bb$^{fl/fl}$*). All mice were given dox.

(**B**) Gross liver images of inducible *Lin28* control mice (N=10) and inducible *Lin28a/Lin28b* DKO mice (N=10) taken after DEN and 12 weeks of CCl_4 treatment. Scale bar = 1 cm.

(**C**) Schema and gross liver images of inducible *Lin28a/Lin28b* control mice (N=6) and inducible *Lin28a/Lin28b* DKO mice (N=6) taken after 12 weeks of CCl_4 treatment. Scale bar = 1 cm.

(**D**) Liver-to-body weight ratios and RT-qPCR analysis assessing inflammation and fibrosis markers for panel C.

(**E**-**F**) Liver bulk tissue of control normal chow diet (NC; N=6) and chronic injury induced from western diet (WD; N=5) were collected for RT-qPCR analysis to assess inflammation and fibrosis markers (**E**) and *Lin28a/b* expression (**F**). PC1 and PC2: *Lin28a/b* expressing liver tumors.

(**G**-**H**) Human samples of normal liver (N=4), stage 4 fibrotic livers (N=12) and *LIN28A/B* expressing tumors (127T) were used for RT-qPCR analysis to assess inflammation and fibrosis markers (**G**) and *LIN28A/B* expression (**H**).

(**I**) Representative IHC of LIN28B expression in human normal liver (N=4), stage 4 fibrotic livers (N=12) and human HCC samples (127T). Scale bar = 50 μm.

(**J**) TCGA analysis shows *LIN28A/B* enrichment in HCCs (LIHC; N=389) compared to normal liver (N=50).

SuppFigure 2.

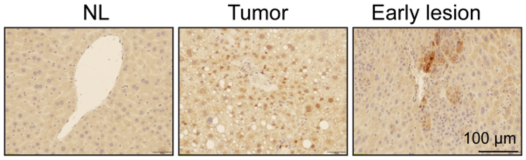

Supplemental Figure 2. DEN tumors and premalignant lesions have increased p-ERK.

Representative IHC images of p-ERK expression in mouse normal liver (N=5), DEN tumors (N=5), and DEN early lesions (N=5). Scale bar = 100 μm.

SuppFigure 3.

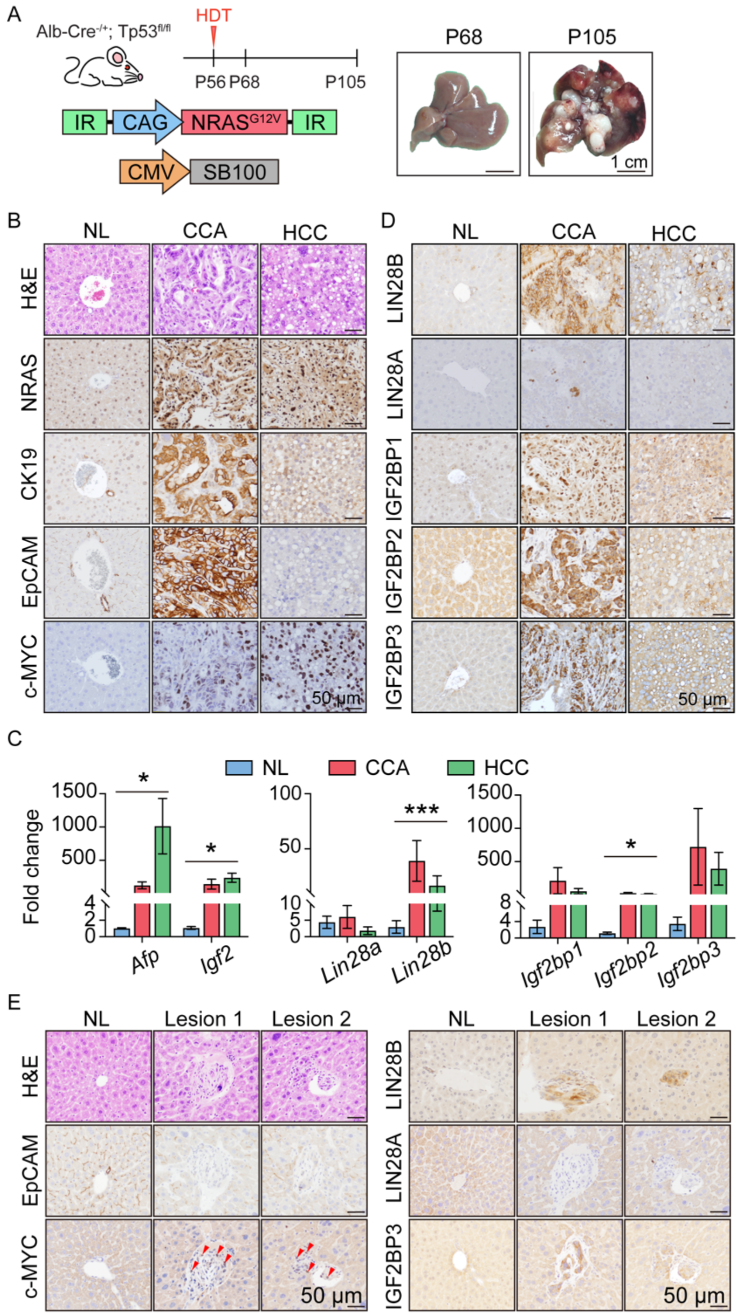

Supplemental Figure 3. *NRAS^{G12V}* overexpression in liver-specific *Tp53* KO mice induced mixed lineage HCCs and CCAs.

(**A**) Overexpression of *NRAS^{G12V}* by hydrodynamic injection (HDT) in *Tp53* KO mice induces the formation of liver tumors. Representative gross images of mice subjected to HDT after 12 (P68) and 49 (P105) days show the kinetics of tumor progression.

(**B**) HCC and CCA histologies were induced by *NRAS^{G12V}* activation, as characterized by H&E or IHC with biliary markers CK19 and EpCAM, or a common liver cancer driver c-MYC. Scale bar = 50 μm.

(**C**) RT-qPCR for *Afp*, *Igf2*, *Lin28a*, *Lin28b*, *Igf2bp1*, *Igf2bp2*, and *Igf2bp3* in normal liver (NL), CCA, and HCC lesions. Tumor samples and adjacent normal liver tissues were collected from 6 individual mice. One-way ANOVA was performed.

(**D**) IHC for LIN28A, LIN28B, IGF2BP1, IGF2BP2 and IGF2BP3 in normal liver (NL), HCC and CAA. Scale bar = 50 μm.

(**E**) H&E shows the morphology of early tumor lesions (age P68, 12 days after HDT) compared to normal liver, and IHC shows EpCAM, c-MYC, LIN28B, LIN28A and IGF2BP3 staining. Red arrows indicate positive staining. Scale bar = 50 μm.

SuppFigure 4.

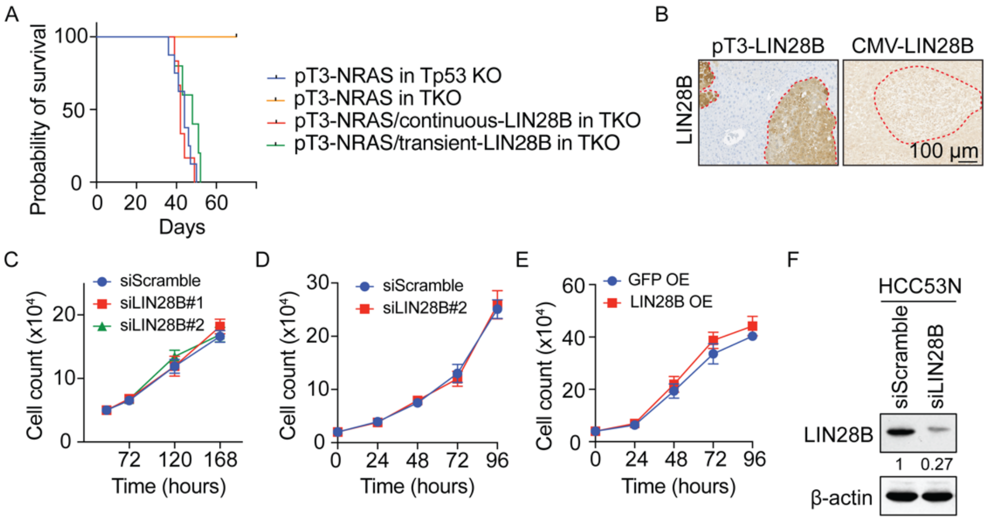

Supplemental Figure 4. LIN28B is not required for the maintenance of *$NRAS^{G12V}$/Tp53* induced HCC.

(**A**) Survival analysis of *Tp53* KO mice (N=5) receiving *$NRAS^{G12V}$*, *Lin28a/Lin28b/Tp53* TKO mice receiving *$NRAS^{G12V}$* (pT3-*$NRAS^{G12V}$* in TKO mice, N=8), *$NRAS^{G12V}$* with *LIN28B* continuous overexpression (pT3-*$NRAS^{G12V}$*/continuous-LIN28B in TKO mice, N=5), or *$NRAS^{G12V}$* with transient *LIN28B* overexpression (pT3-*$NRAS^{G12V}$*/transient-LIN28B, N=5).

(**B**) LIN28B IHC in *$NRAS^{G12V}$* driven tumors rescued by the integrating *pT3-LIN28B* transposon plasmid or the non-integrating *pCMV-LIN28B* plasmid. Scale bar = 100 μm.

(**C**) Growth analysis of Huh7 cells treated with siLIN28B#1 or siLIN28B#2 compared to siScramble (N=3).

(**D**) Growth analysis of HCC53N cells treated with siLIN28B compared to siScramble (N=3).

(**E**) Growth analysis of SNU308 cells overexpressing LIN28B compared to GFP (N=3).

(**F**) Western blot analysis for LIN28B expression in HCC53N cells treated with siLIN28B compared to siScramble. Numbers below the box show relative band intensity.

SuppFigure 5.

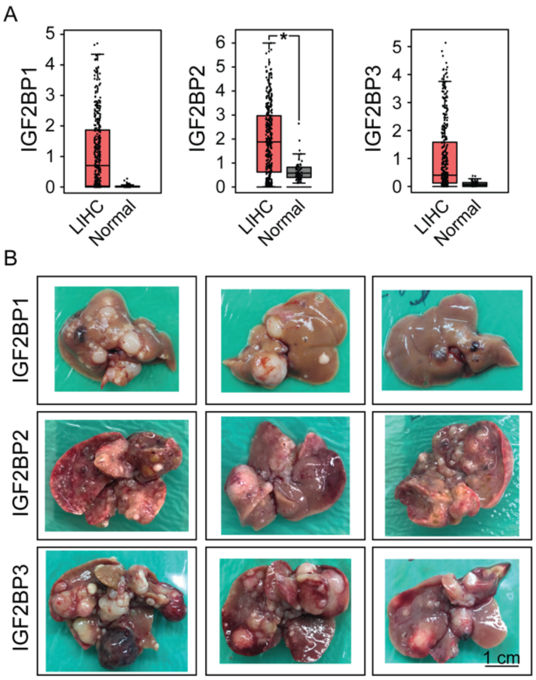

Supplemental Figure 5. *Igf2bp1*, *Igf2bp2*, and *Igf2bp3* can rescue HCCs in *Lin28a/Lin28b/Tp53* TKO mice.

(**A**) TCGA analysis shows *IGF2BP1/2/3* overexpression in HCC (LIHC; N=389) compared to normal liver (N=50). One-way ANOVA was used to assess statistical differences.

(**B**) Gross liver images of *Lin28a/Lin28b/Tp53* TKO mice injected with transposons carrying *Igf2bp1* (N=3), *Igf2bp2* (N=3) or *Igf2bp3* (N=3) for 7 weeks. Scale bar = 1 cm.

SuppFigure. 6

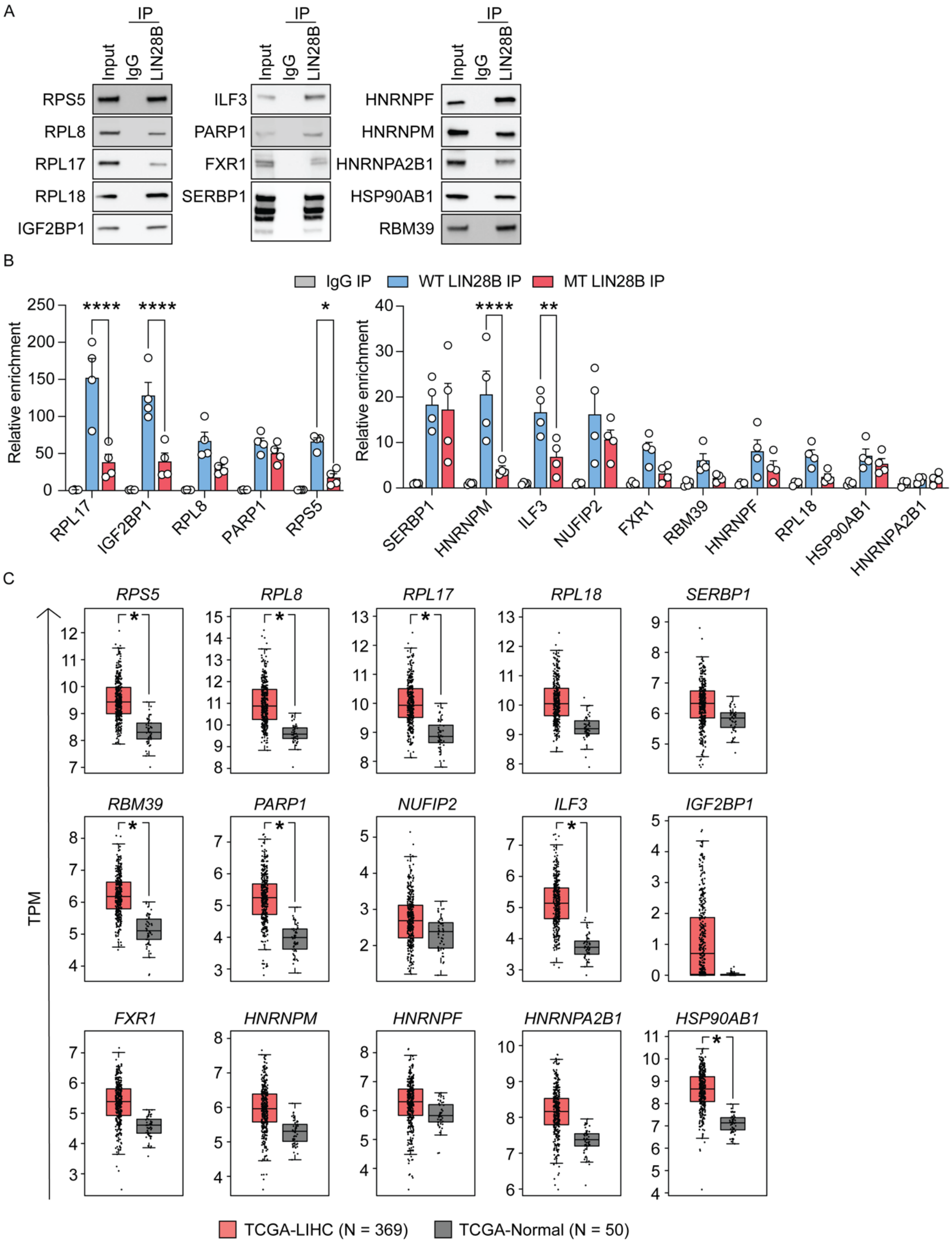

Supplemental Figure 6. LIN28 targets interact with LIN28 as RNAs and proteins and are upregulated in human HCCs.

(**A**) Immunoprecipitation followed by western blot analysis shows that 14 factors bound to LIN28B in Huh7 cells.

(**B**) RIP-qPCR analysis shows the interaction of 15 LIN28B mRNA targets to WT LIN28B versus mutant LIN28B protein with an early termination that leads to the loss of zinc knuckle domains (N=3).

(**C**) TCGA analysis showed that 15 LIN28 RBP targets analyzed in this paper are enriched in human HCC samples (LIHC; N=389) compared to normal liver tissues (N=50). One-way ANOVA was performed.

.

SuppFigure 7.

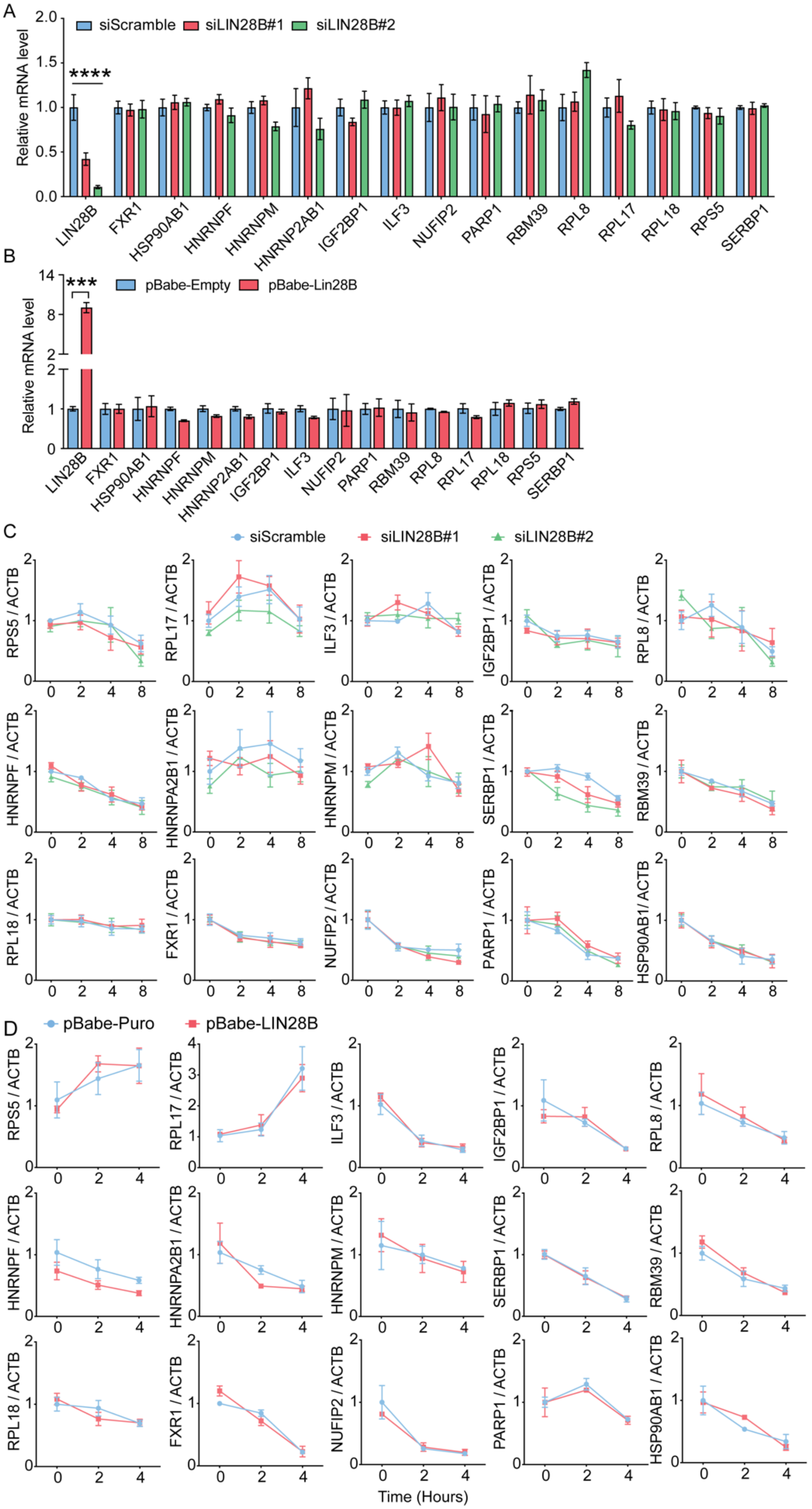

Supplemental Figure 7. LIN28B does not regulate candidate RBP targets through mRNA transcription or stabilization.

(**A**-**B**) RT-qPCR analysis of mRNA levels of LIN28 targets 48 hours after *LIN28B* siRNA knockdown in Huh7 cells (N=3-4) (**A**) and 72 hours after *LIN28B* overexpression in SNU308 cells (N=3) (**B**). One-way ANOVA was performed.

(**C**-**D**) Measurements of RNA stability using RT-qPCR analysis quantifying mRNA levels at 0, 2, 4 and 8 hours after actinomycin D treatment in *LIN28B* knockdown Huh7 cells (N=3) (**C**) and *LIN28B* overexpressing SNU308 cells (N=3) (**D**).

SuppFigure 8.

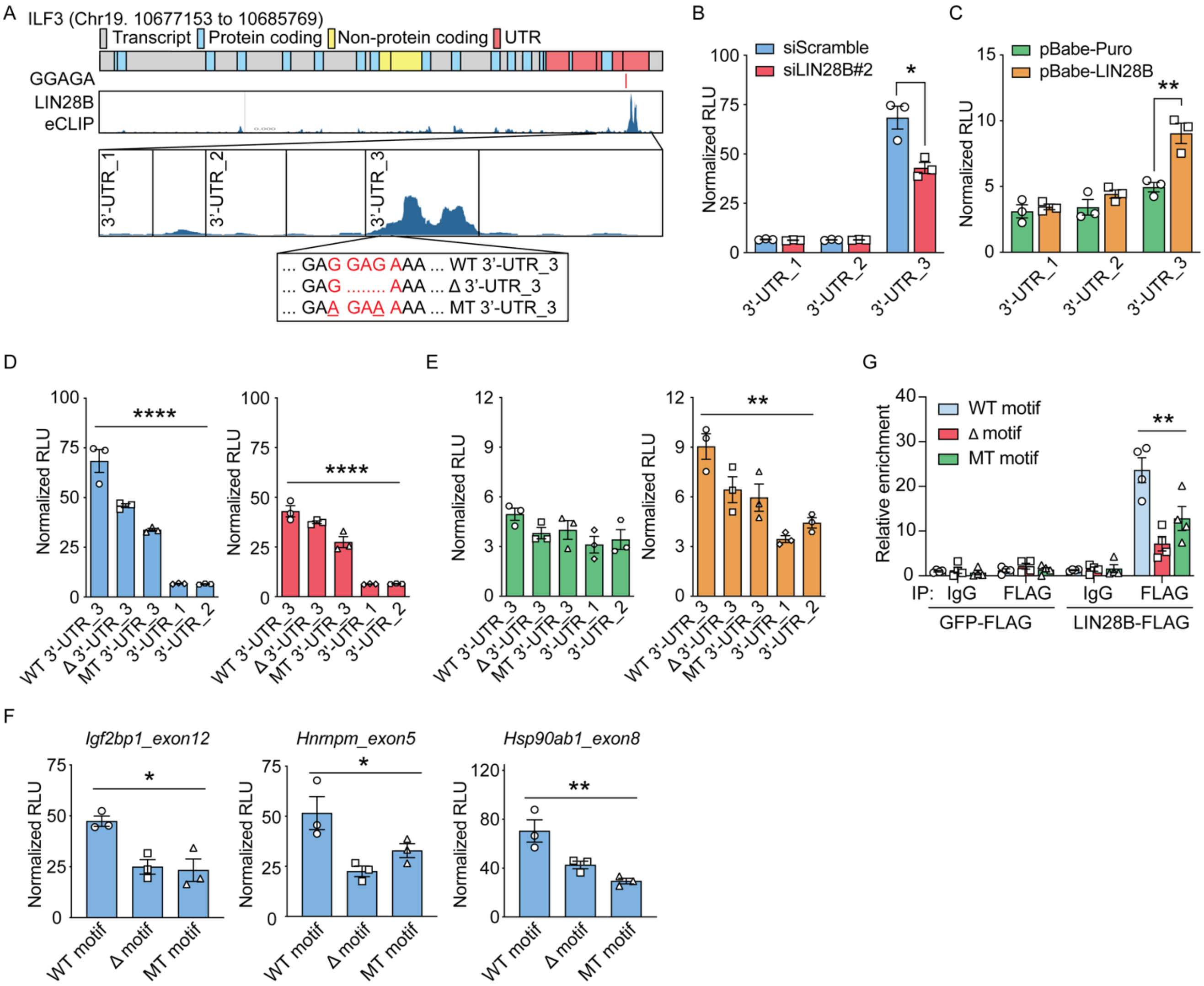

Supplemental Figure 8. LIN28B regulates *ILF3* translation through direct mRNA binding.

(**A**) eCLIP data shows LIN28B binding regions on *ILF3* mRNA. The red marks under the gene indicate the location of consensus LIN28B binding motifs (GGAGA). Deletion (Δ) and mutation (MT) of consensus motifs were introduced to prevent LIN28B binding. All sequences were inserted into the Renilla 3'UTR region.

(**B**) Renilla luciferase activity promoted by *ILF3* 3'UTR_1/2/3 reporters in LIN28B siRNA knockdown (N=3) vs. control Huh7 cells (N=3).

(**C**) Renilla luciferase activity promoted by *ILF3* 3'UTR_1/2/3 reporters in LIN28B overexpression (N=3) vs. control SNU308 cells (N=3).

(**D**-**E**) Renilla luciferase activity promoted by WT *ILF3* sequences compared to deletion (Δ) and mutation (MT) containing reporters in control (blue) and LIN28B siRNA knockdown (red) Huh7 cells (N=3) (**D**), and in control (green) and LIN28B overexpression (orange) SNU308 cells (N=3) (**E**). One-way ANOVA was performed.

(**F**) Renilla luciferase activity promoted by WT binding motif, deletion (Δ) and mutation (MT) motif of Igfbp2, Hnrnpm and Hsp90ab1 (N=3). One-way ANOVA was performed.

(**G**) RIP-qPCR examine luciferase enrichment from 293T cells transfected with GFP-FLAG or LIN28B-FLAG along with equal amount of luciferase promoted by the WT, deletion (Δ motif), and mutant (MT motif) ILF'3 sequence (N=3). One-way ANOVA was performed.

SuppFigure 9.

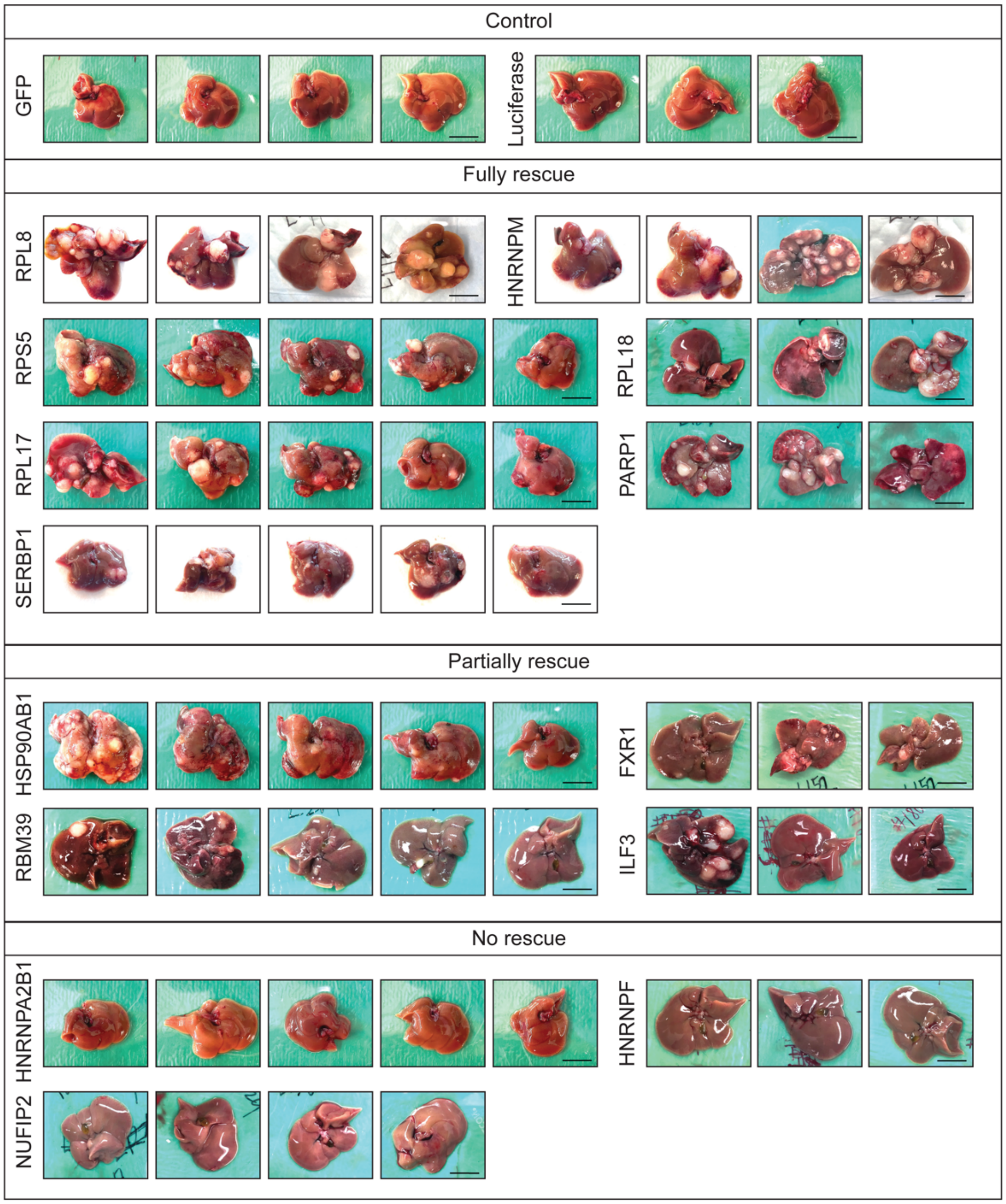

Supplemental Figure 9. Lin28 target genes can rescue tumorigenesis in *Lin28a/Lin28b/Tp53* TKO mice.

All gross liver images of *Lin28a/Lin28b/Tp53* TKO mice receiving $NRAS^{G12V}$ in combination with *pT3-RBPs* (N>3), *pT3-eGFP* (N=4), or *pT3-Luciferase* (N=3) for 7 weeks. IGF2BP1 is shown in Supplemental Figure 6B. Scale bar = 1 cm.

SuppFigure 10.

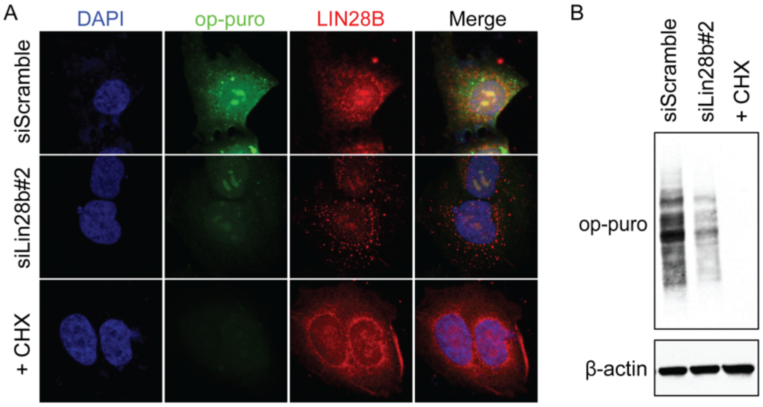

Supplemental Figure 10. *LIN28B* knockdown downregulates translation.

(**A**-**B**) Translation capacity assessed with immunocytochemistry (**A**) and western blot analysis of OP-puro in Huh7 cells with *LIN28B* knockdown (siLin28b#2) compared to control (siScramble) (**B**). Treatment of cycloheximide (CHX) in parental cells was used as a control to block translation.
