# Supplementary material for: Liver cancer initiation requires translational activation by an oncofetal regulon involving LIN28 proteins
Source: J Clin Invest. 2024 Jun 13;134(15):e165734. doi: 10.1172/JCI165734 (PMC11290964; doi:10.1172/JCI165734)

Blot and gel image

Unedited blot images for Figure 4B.

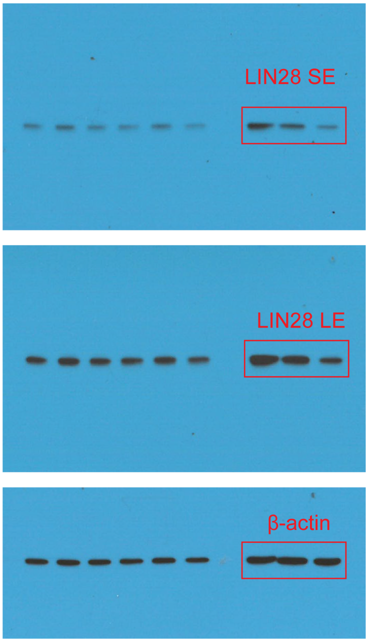

Unedited blot images for Figure 5B.

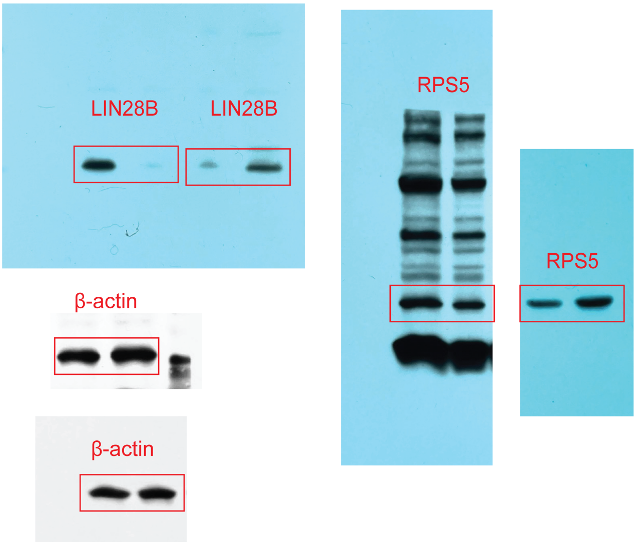

Unedited blot images for Figure 6D.

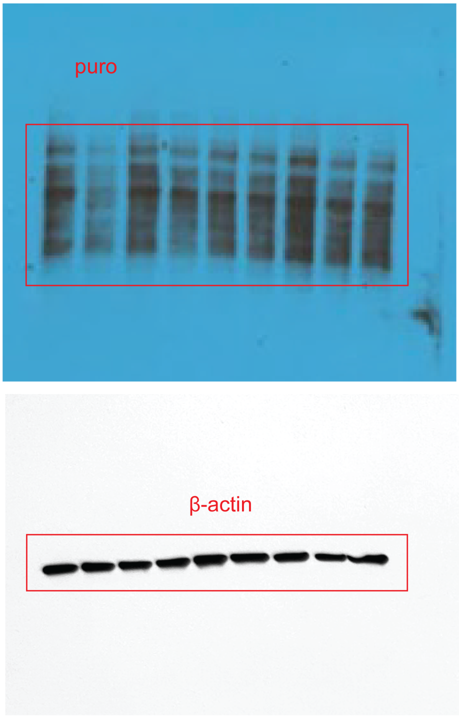

Unedited gel images for Supplemental Figure 1A.

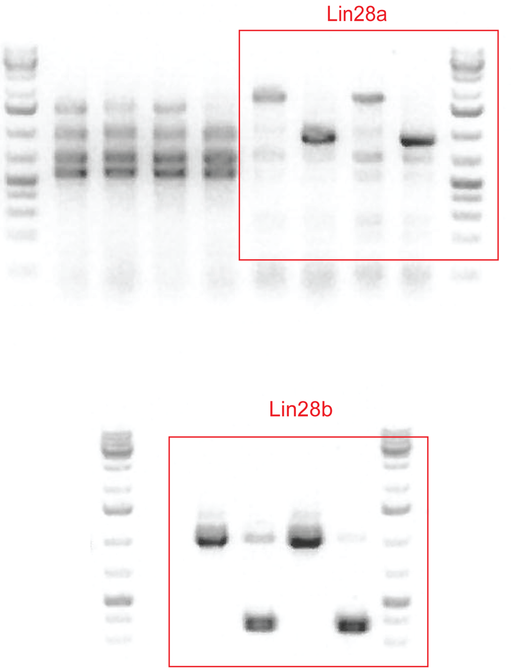

Unedited blot images for Supplemental Figure 4F.

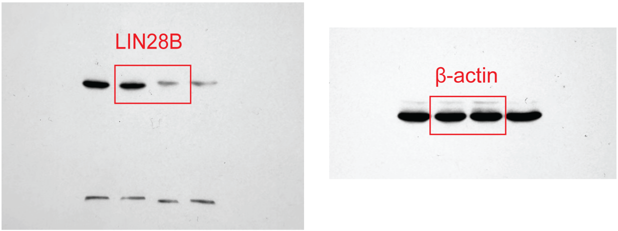

Unedited blot images for Supplemental Figure 6A.

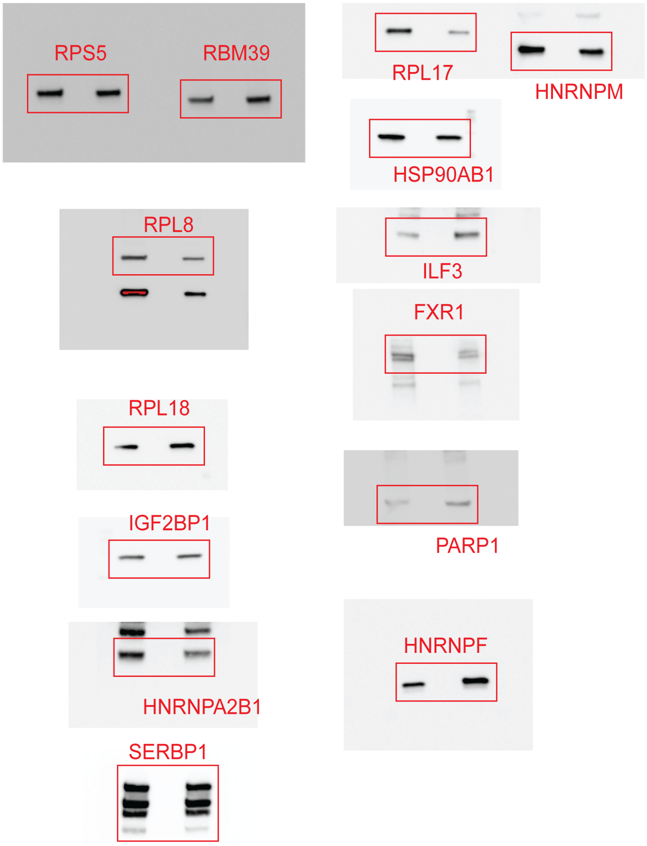

Unedited blot images for Supplemental Figure 10B.

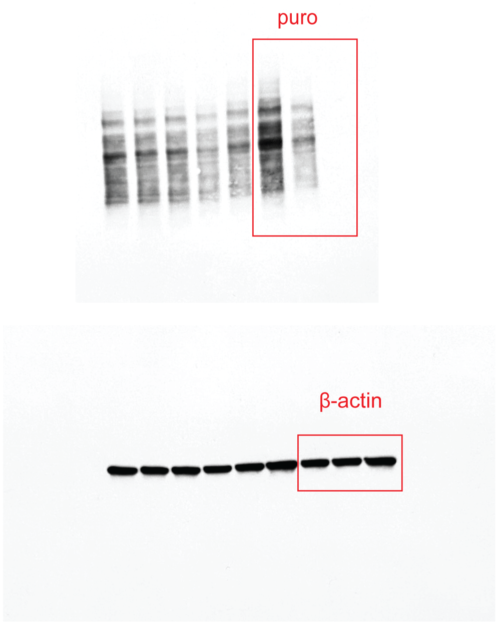

Supplement: Unedited blot and gel images [file jci-134-165734-s147.pdf]
